# Maturation and circuit integration of transplanted human cortical organoids

**DOI:** 10.1038/s41586-022-05277-w

**Published:** 2022-10-12

**Authors:** Omer Revah, Felicity Gore, Kevin W. Kelley, Jimena Andersen, Noriaki Sakai, Xiaoyu Chen, Min-Yin Li, Fikri Birey, Xiao Yang, Nay L. Saw, Samuel W. Baker, Neal D. Amin, Shravanti Kulkarni, Rachana Mudipalli, Bianxiao Cui, Seiji Nishino, Gerald A. Grant, Juliet K. Knowles, Mehrdad Shamloo, John R. Huguenard, Karl Deisseroth, Sergiu P. Pașca

**Affiliations:** 1 Department of Psychiatry and Behavioral Sciences, Stanford University, Stanford, CA USA; 2 Stanford Brain Organogenesis, Wu Tsai Neurosciences Institute and Bio-X, Stanford University, Stanford, CA USA; 3 Department of Bioengineering, Stanford University, Stanford, CA USA; 4 Department of Chemistry, Stanford University, Stanford, CA USA; 5 Stanford Behavioral and Functional Neuroscience Laboratory, Wu Tsai Neurosciences Institute, Stanford University, Stanford, CA USA; 6 Department of Comparative Medicine, Stanford University, Stanford, CA USA; 7 Department of Neurosurgery, Stanford University, Stanford, CA USA; 8 Department of Neurology and Neurological Sciences, Stanford, CA USA; 9 Howard Hughes Medical Institute, Stanford University, Stanford, CA USA

**Keywords:** Neuroscience, Developmental biology

## Abstract

Self-organizing neural organoids represent a promising in vitro platform with which to model human development and disease^1–5^. However, organoids lack the connectivity that exists in vivo, which limits maturation and makes integration with other circuits that control behaviour impossible. Here we show that human stem cell-derived cortical organoids transplanted into the somatosensory cortex of newborn athymic rats develop mature cell types that integrate into sensory and motivation-related circuits. MRI reveals post-transplantation organoid growth across multiple stem cell lines and animals, whereas single-nucleus profiling shows progression of corticogenesis and the emergence of activity-dependent transcriptional programs. Indeed, transplanted cortical neurons display more complex morphological, synaptic and intrinsic membrane properties than their in vitro counterparts, which enables the discovery of defects in neurons derived from individuals with Timothy syndrome. Anatomical and functional tracings show that transplanted organoids receive thalamocortical and corticocortical inputs, and in vivo recordings of neural activity demonstrate that these inputs can produce sensory responses in human cells. Finally, cortical organoids extend axons throughout the rat brain and their optogenetic activation can drive reward-seeking behaviour. Thus, transplanted human cortical neurons mature and engage host circuits that control behaviour. We anticipate that this approach will be useful for detecting circuit-level phenotypes in patient-derived cells that cannot otherwise be uncovered.

## Abstract (web summary)

Human stem cell-derived cortical organoids transplanted into rats mature and integrate into sensory and motivation circuits to influence behaviour.

## Main

Human brain development is a remarkable self-organizing process in which cells proliferate, differentiate, migrate and wire to form functioning neural circuits that are subsequently refined by sensory experience^1^. A critical challenge for understanding human brain development, particularly in the context of disease, is a lack of access to brain tissue. By applying instructive signals to human induced pluripotent stem (hiPS) cells grown in three-dimensional (3D) cultures, self-organizing organoids resembling specific regions of the nervous system, including human cortical organoids (hCO; also known as human cortical spheroids) can be generated^2–6^. However, there are several limitations that restrict their broader applications in understanding neural circuit development and function. Specifically, it is unclear whether maturation of hCO is constrained by the lack of certain microenvironments and sensory inputs that exist in vivo. Moreover, as hCO are not integrated into circuits that can generate behavioural outputs, their utility in modelling genetically complex and behaviourally defined neuropsychiatric diseases is currently limited.

Transplantation of hCO into intact living brains has the potential to overcome these limitations. Previous studies have demonstrated that human neurons transplanted into the rodent cortex survive, project and make connections with rodent cells^7–12^. However, these experiments have typically been performed in adult animals, which probably limits synaptic and axonal integration. Here we introduce a transplantation paradigm in which we transplanted 3D hCO derived from hiPS cells into the primary somatosensory cortex (S1) of immunodeficient rats at an early, plastic developmental stage^13^. Neurons from transplanted hCO (t-hCO) undergo substantial maturation, receive thalamocortical and corticocortical inputs that are capable of evoking sensory responses and extend axonal projections into the rat brain that can drive reward-seeking behaviours. Advanced maturation in t-hCO reveals defects in neurons derived from patients with Timothy syndrome (TS), a severe genetic disease caused by a mutation in the L-type voltage-sensitive calcium channel Ca_V_1.2 (encoded by *CACNA1C*)^14^.

**Fig. 1:**
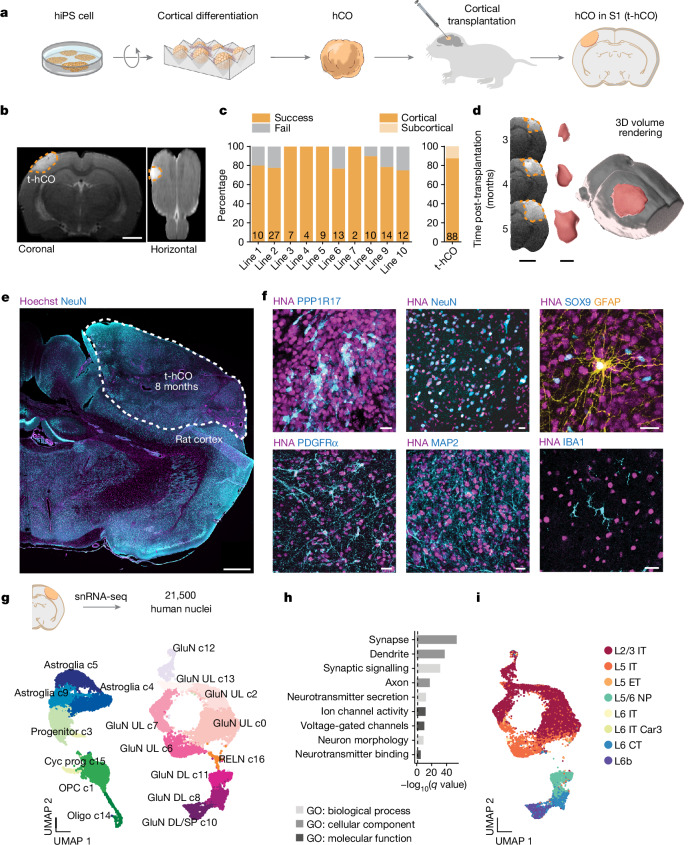
Transplantation of human cortical organoids in the developing rat cortex. **a**, Schematic of the experimental design. hCO generated from hiPS cells are transplanted at days 30–60 of differentiation into the S1 of newborn athymic rats. **b**, Coronal and horizontal view T2-weighted MRI images showing t-hCO in the S1 at 2 months post-transplantation. Scale bar, 2 mm. **c**, Quantification of the success rate of transplantations shown per hiPS cell line (*n* = 108, numbers inside bars indicate number of t-hCO per hIPS cell line) and cortical or subcortical position (*n* = 88). **d**, Coronal MRI images (left; scale bar, 3 mm) and corresponding 3D volume reconstructions (scale bar, 3 mm) showing t-hCO growth over 3 months. **e**, Overview of example t-hCOs in the rat cortex. Scale bar, 1 mm. **f**, Representative immunocytochemistry images of t-hCO showing from top left to right (at time in differentiation): PPP1R17 (4 months), NeuN (8 months), SOX9 and GFAP (8 months), PDGFRα; (8 months), MAP2 (8 months) and IBA1 (8 months). Scale bars, 20 μm. Co-expression of HNA indicates cells of human origin. **g**, snRNA-seq: uniform manifold approximation and projection (UMAP) dimensional reduction visualization of all clustered high-quality t-hCO nuclei after Seurat integration (*n* = 3 t-hCO samples, *n* = 2 hiPS cell lines). Astroglia, astrocyte lineage cells; cyc prog, cycling progenitor; GluN DL, deep layer glutamatergic neuron; GluN DL/SP, deep layer and subplate glutamatergic neuron; GluN UL, upper layer glutamatergic neuron; oligo, oligodendrocyte; OPC, oligodendrocyte progenitor cell; RELN, reelin neurons. **h**, Gene Ontology (GO) term enrichment analysis of genes significantly upregulated (adjusted *P* < 0.05, fold change > 2, expressed in at least 10% of nuclei) in t-hCO glutamatergic neurons compared with hCO glutamatergic neurons. The dashed line denotes a *q* value of 0.05. **i**, UMAP visualization of GluN cell types of t-hCO using label transfer from the adult human motor cortex^22^ snRNA-seq reference dataset. CT, corticothalamic cell; ET, extratelencephalic cell; IT, intratelencephalic cell; NP, near-projecting.

**Extended Data Fig. 1:**
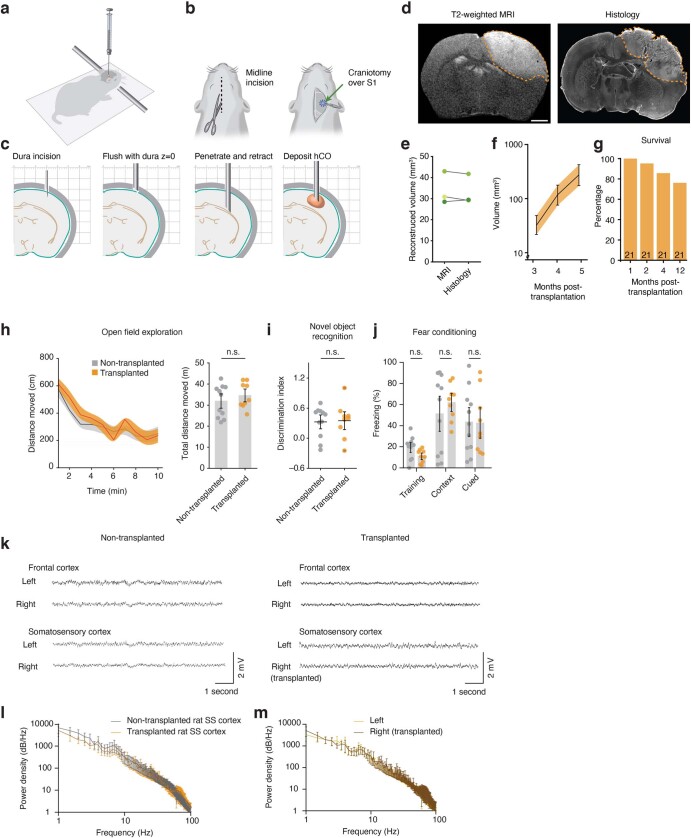
Schematic of the transplantation procedure and effects on animal behaviour. **a–c**. Schematics of surgical approach. **d**. *Left*, t-hCO visualized with T2-weighted MRI. *Right*, t-hCO visualized with DAPI. **e**. Reconstructed volumes of the same t-hCO calculated from MRI or histological slices (n = 3, P = 0.9160 t-hCO). **f**. Quantification of MRI volume reconstruction over time (n = 6 t-hCO, *P = 0.0239). **g**. Quantification of survival in transplanted animals over time. **h–j**. Animals transplanted with t-hCO do not show behavioral deficits or seizures. **h**. Distance traveled in open field arena by transplanted (orange) or non-transplanted control animals (grey) for each minute of a 10-minute testing session (left) and across the entire 10-minute testing period (right) (n = 11 non-transplanted control rats, 9 transplanted rats, P = 0.4082). **i**. Discrimination index ((time spent interacting with novel object - time spent interacting with familiar object)/(time spent interacting with novel object + time spent interacting with familiar object)) during novel object test calculated for transplanted (orange) or non-transplanted control animals (grey) (n = 11 non-transplanted control rats, 9 transplanted rats, P = 0.8756). **j**. Freezing behaviour during fear conditioning training, contextual fear memory test, and cued fear memory test for transplanted (orange) or non-transplanted control animals (grey) (n = 11 non-transplanted control rats, 9 transplanted rats, P = 0.9599). **k**. Representative EEG recordings from the frontal and somatosensory cortices of non-transplanted (left) and transplanted (right) rats. **l**. Power spectral density plots of EEG activity recorded in the somatosensory cortex of non-transplanted control and transplanted rats (n = 3 transplanted rats, 3 non-transplanted rats). **m**. Power spectral density plots of somatosensory cortex EEG activity recorded simultaneously in the non-transplanted and transplanted hemisphere of individual rats (n = 3 transplanted rats). Data are presented as mean ± SEM.

**Extended Data Fig. 3:**
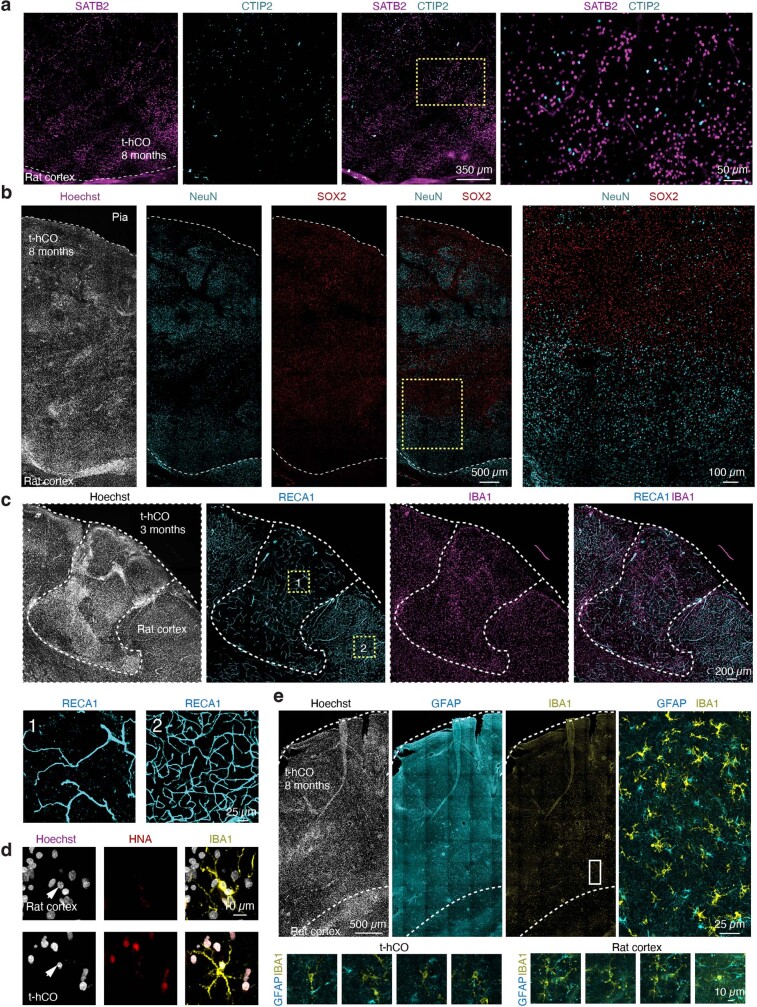
Immunohistochemical characterization of t-hCO continued. **a**. Representative images of SATB2 and CTIP2 expression in t-hCO. **b**. Example images of SOX2 and NeuN expression in t-hCO. **c**. Representative images of rat-edothelial-marker-1 (RECA1) and IBA1 expression in t-hCO and nearby rat cortex 3 months after transplantation. **d**. HNA and IBA1 expression in t-hCO. Absence of overlap between HNA and IBA1 suggests microglia originate from rat. **e**. GFAP and IBA1 expression in t-hCO and rat cortex. All images were acquired 7–9 months in differentiation unless otherwise stated.

To study human-derived cortical neurons within in vivo circuits, we stereotactically transplanted intact, 3D hCO into the S1 of early postnatal athymic rats (postnatal days 3–7) (Fig. 1a and Extended Data Fig. 1a–c). At this time point, thalamocortical and corticocortical axonal projections have not yet completed their innervation of the S1 (ref. ^13^). This approach therefore aims to maximize integration of t-hCO while minimally compromising endogenous circuitry. To visualize the location of t-hCO in living animals, we performed T2-weighted MRI reconstructions of the rat brain at 2–3 months post-transplantation (Fig. 1b and Extended Data Fig. 1d). t-hCO were readily observed and volume measurements of t-hCO were similar to those calculated from fixed slices (Extended Data Fig. 1d,e; *P* > 0.05). We identified t-hCO in 81% of engrafted animals at approximately 2 months post-transplantation (*n* = 72 animals; hCO from 10 hiPS cell lines; hiPS cell lines are listed in Supplementary Table 1). Of these, 87% were located in the cerebral cortex (Fig. 1c). By performing consecutive MRI scans at multiple time points in the same transplanted rats, we found that t-hCO increased ninefold in volume over 3 months (Fig. 1d and Extended Data Fig. 1f). The survival rate of transplanted animals was high 12 months after transplantation (74%) (Extended Data Fig. 1g and Supplementary Table 2), and no discernible locomotor or memory deficits, gliosis or electroencephalogram (EEG) abnormalities were detected (Extended Data Figs. 1h–m and 3e).

**Extended Data Fig. 4:**
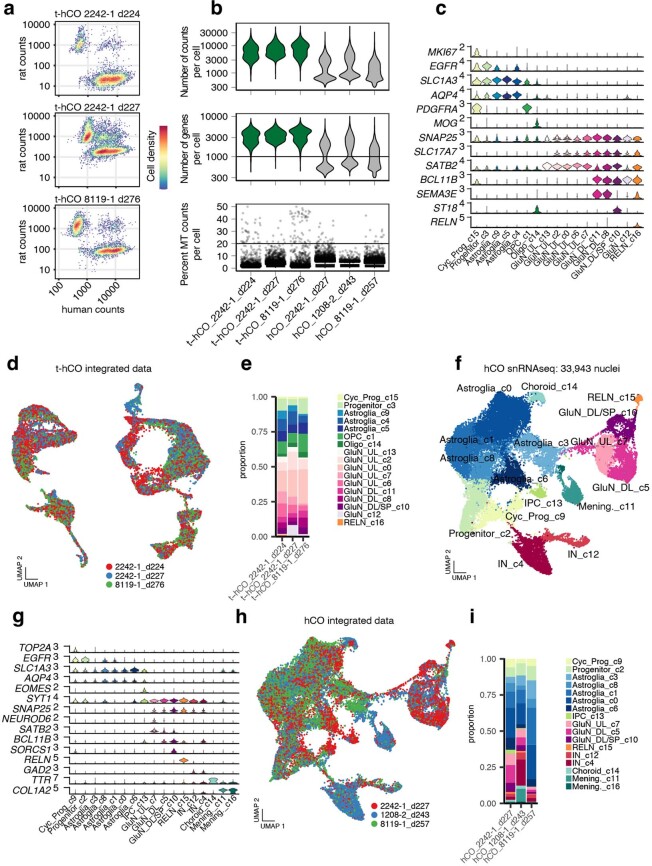
Data quality of single nucleus RNA-seq samples and hCO analysis. **a**. The number of snRNA-seq read counts aligned to rat and human genome for each nucleus split by sample. Human nuclei were defined as nuclei with >95% of total reads aligning to the human genome. **b**. snRNA-seq quality metrics showing the distribution of the number of counts, number of genes, and mitochondrial (MT) gene fraction per cell in each sample. MT gene fraction plotted as boxplots (horizontal line denotes median; lower and upper hinges correspond to the first and third quartiles; whiskers extend 1.5 times the interquartile range with outliers shown outside this range). Lines denote nuclei quality thresholds. **c**. Gene expression violin plots for selected marker genes in t-hCO. **d**. Same integrated UMAP as shown in Fig. 1g, colored by t-hCO sample. **e**. Cell type proportions across t-hCO samples colored by clusters. **f**. UMAP dimensional reduction visualization of all clustered high-quality hCO nuclei after Seurat integration (n = 3 t-hCO samples from 3 hiPS lines). **g**. Gene expression violin plots for selected marker genes. **h**. Same integrated UMAP as shown in panel f, colored by hCO sample. **i**. Cell type proportions across hCO samples colored by clusters. hCO and t-hCO from 2242-1 at day 227 are taken from the same differentiation batch maintained in parallel. Cyc. prog., cycling progenitor; Astroglia, astrocyte lineage cell; IPC, intermediate progenitor cell; GluN_UL, upper layer glutamatergic neuron; GluN_DL, deep layer glutamatergic neuron; GluN_DL/SP, deep layer and subplate glutamatergic neurons; RELN, Reelin neurons; IN, GABAergic neurons; Choroid, choroid plexus-like cells; Mening., meningeal-like cells.

We next assessed the cytoarchitecture and gross cellular composition of t-hCO. Antibody staining for rat endothelia revealed vascularization of t-hCO, whereas staining for IBA1 revealed the presence of rat microglia throughout the graft (Fig. 1f and Extended Data Fig. 3c,d). Immuno-stainings identified human nuclear antigen (HNA)-positive cells that co-expressed PPP1R17 (cortical progenitors), NeuN (neurons), SOX9 and GFAP (glial lineage cells) or PDGFRα (oligodendrocyte progenitor cells) (Fig. 1f). To explore the cellular composition of t-hCO with single-cell resolution, we performed single-nucleus RNA sequencing (snRNA-seq) at approximately 8 months of differentiation. Quality filtering and removal of rat nuclei yielded 21,500 high-quality human single-nucleus profiles (Fig. 1g and Extended Data Fig. 4a,b). Expression patterns of canonical cell-type markers identified clusters of major cortical cell classes, including both deep and superficial layer glutamatergic neurons, cycling progenitors, oligodendrocytes and astrocyte lineage cells (Fig. 1g, Extended Data Fig. 4c and Supplementary Table 3). Immunostainings for SATB2 and CTIP2 revealed that, despite the presence of cortical layer subtypes, t-hCO displayed no obvious anatomical lamination (Extended Data Fig. 3a). snRNA-seq of stage-matched hCO yielded broadly similar cell classes with some exceptions, including a lack of oligodendrocytes and the presence of GABAergic neurons, which may reflect in vitro conditions that favour the generation of some ventral progenitors in long-term cultures, as previously reported^15^ (Extended Data Fig. 4f–i and Supplementary Table 4). Differential gene expression analysis highlighted substantial differences in glutamatergic neurons between t-hCO and hCO (Supplementary Table 5), including upregulation of gene sets associated with neuronal maturation, such as synaptic signalling, dendrite localization and voltage-gated channel activity (Fig. 1h and Supplementary Table 6). Thus, t-hCO cortical glutamatergic neurons display advanced transcriptional maturation.

**Fig. 2:**
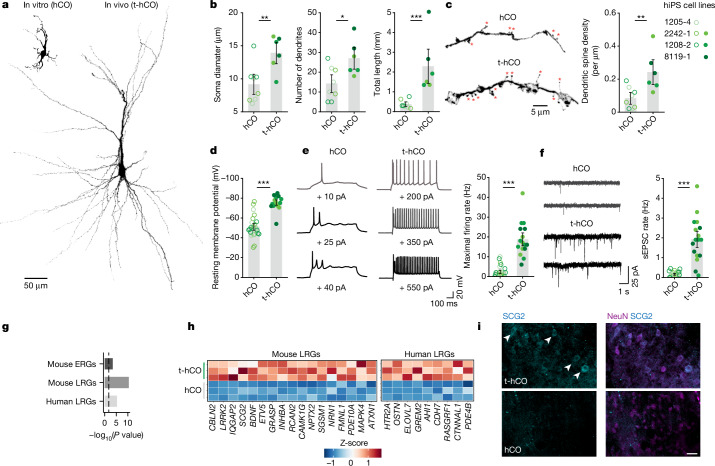
t-hCO neurons undergo advanced maturation. **a**, 3D reconstruction of biocytin-filled hCO and t-hCO neurons at 8 months of differentiation. **b**, Quantification of morphological features (*n* = 8 hCO neurons, *n* = 6 t-hCO neurons; **P = 0.0084, *P = 0.0179 and ***P < 0.0001). **c**, 3D-reconstructed dendritic branches of hCO and t-hCO at 8 months of differentiation. The red asterisks indicate putative dendritic spines. Quantification of dendritic spine density (*n* = 8 hCO neurons, *n* = 6 t-hCO neurons; **P = 0.0092). **d**, Quantification of the resting membrane potential (*n* = 25 hCO neurons, *n* = 16 t-hCO neurons; ***P < 0.0001). **e**, Repetitive action potential firing in hCO and t-hCO induced by increasing current injections, and quantification of the maximal firing rate (*n* = 25 hCO neurons, *n* = 16 t-hCO neurons; ***P < 0.0001). **f**, Spontaneous EPSCs (sEPSCs) in hCO and t-hCO neurons at 8 months of differentiation, and quantification of the frequency of synaptic events (*n* = 25 hCO neurons, *n* = 17 t-hCO neurons; ***P < 0.0001). For **b**–**f**, hCO and t-hCO from line 1208-2 are taken from the same differentiation batch maintained in parallel. **g**, Gene set enrichment analysis (one-sided Fisher’s exact test) of genes significantly upregulated (adjusted *P* < 0.05, fold change > 2, expressed in at least 10% of nuclei) in t-hCO glutamatergic neurons compared with hCO glutamatergic neurons with gene sets of both early-response (ERG) and late-response (LRG) activity-dependent genes identified from an in vivo mouse study^16^ and human-specific LRGs from in vitro neurons^17^. The dashed line denotes Bonferroni-corrected *P* value of 0.05. **h**, GluN gene expression (pseudobulk and scaled for each gene) across snRNA-seq replicates of LRG genes significantly upregulated in t-hCO glutamatergic neurons. **i**, Immunostaining showing SCG2 expression in t-hCO (top) and hCO (bottom) neurons. White arrowheads indicate SCG2^+^ cells. Scale bar, 25 µm. Data are presented as mean ± s.e.m.

To examine whether these transcriptional changes in t-hCO are associated with morphological differences between in vitro hCO and in vivo t-hCO, we reconstructed stage-matched biocytin-filled hCO and t-hCO neurons in acute slices at 7–8 months of differentiation (Fig. 2a). t-hCO neurons were considerably larger with the soma 1.5-fold larger in diameter, twofold more dendrites and, overall, a sixfold increase in total dendrite length than hCO in vitro (Fig. 2b). Moreover, we observed significantly higher dendritic spine density in t-hCO neurons than in hCO neurons (Fig. 2c). This suggests that t-hCO neurons undergo extensive dendritic extension and arborization, which in combination with ongoing cellular proliferation probably contributes to the extensive growth of t-hCO following transplantation (Fig. 1d and Extended Data Fig. 1f). This prompted us to investigate electrophysiological properties. The membrane capacitance was eightfold higher (Extended Data Fig. 8d), the resting membrane potential was more hyperpolarized (by approximately 20 mV) and current injections elicited higher maximal firing rates in t-hCO neurons than in hCO neurons in vitro (Fig. 2d,e), consistent with the larger and more complex morphological features of t-hCO. Furthermore, the rate of spontaneous excitatory postsynaptic current events (EPSCs) in t-hCO neurons was significantly higher (Fig. 2f), indicating that the increase in dendritic spine density observed in t-hCO neurons is associated with an increase in the number of functional excitatory synapses. We confirmed the immature profile of hCO neurons in vitro by recording from labelled glutamatergic neurons (Extended Data Fig. 6a–c).

In accordance with the increased activity observed in t-hCO in ex vivo slices, snRNA-seq revealed upregulation of activity-dependent gene transcripts in t-hCO compared with hCO in vitro. t-hCO glutamatergic neurons expressed higher levels of late-response activity-regulated genes (Fig. 2g,h) found in previous studies of mouse and human neurons^16,17^. For example, *BDNF*^18^, *SCG2* and *OSTN*, a primate-specific activity-regulated gene^17^, showed increased expression in t-hCO neurons compared with hCO neurons (Fig. 2g–i). Therefore, across transcriptional, morphological and functional analyses, t-hCO neurons displayed features of enhanced maturation compared with hCO neurons.

**Fig. 3:**
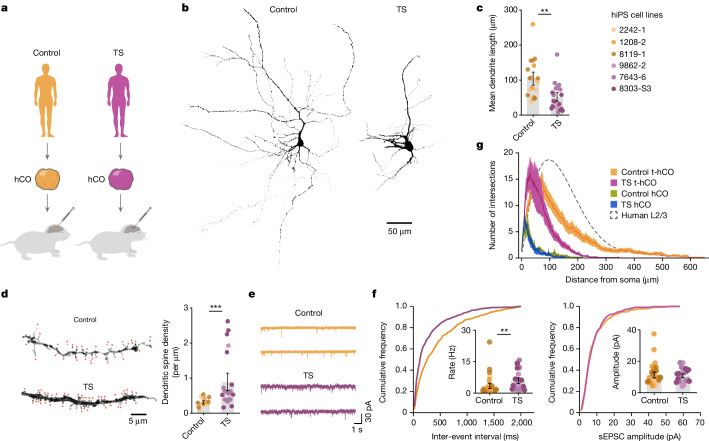
Advanced neuronal features in t-hCO reveal activity-dependent disease phenotypes in human cortical neurons. **a**, Transplantation of hCO generated from control and TS hiPS cell lines into newborn rats. **b**, 3D reconstruction of biocytin-filled t-hCO neurons at 8 months of differentiation. **c**, Quantification of mean dendrite length (*n* = 19 control neurons, *n* = 21 TS neurons; **P = 0.0041). **d**, 3D-reconstructed dendritic branches from control and TS t-hCO at 8 months of differentiation, and quantification of dendritic spine density (*n* = 16 control neurons, *n* = 21 TS neurons, ***P < 0.0001). Red asterisks indicate putative dendritic spines. **e**, Spontaneous EPSCs in control and TS t-hCO neurons at 8 months of differentiation. **f**, Cumulative frequency plots and quantification of synaptic event frequency and amplitude (*n* = 32 control neurons, *n* = 26 TS neurons; **P = 0.0076 and *P* = 0.8102). **g**, Sholl analysis of TS and control neurons in hCO and t-hCO. The dashed line shows postnatal human L2/3 pyramidal neurons for comparison (*n* = 24 control t-hCO neurons, *n* = 21 TS t-hCO neurons, *n* = 8 control hCO neurons and *n* = 7 TS hCO neurons). Data are presented as mean ± s.e.m.

**Extended Data Fig. 5:**
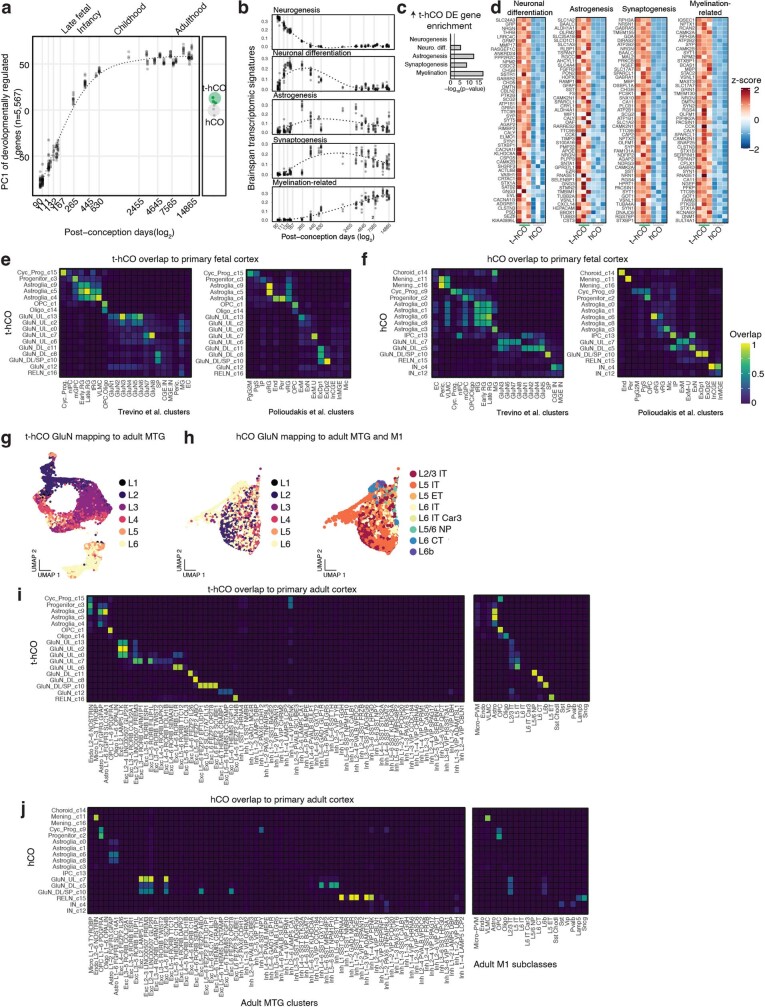
Transplanted hCO RNA-seq comparisons to fetal and adult human cortex. **a**. Left: The first principal component (PC1) calculated on gene expression (log base 2 RPKM, reads per kilobase of exon per million reads mapped) from human cortical BrainSpan samples^23^ and pseudobulk (Methods) snRNA-seq samples using previously defined developmentally regulated genes^38^. Right: PC1 values for t-hCO and hCO samples. **b**. The sample weights (Methods) of five neurodevelopmental transcriptomic signatures identified by Zhu et al.^38^ across human cortical BrainSpan samples. Dashed lines denote fitted curves by LOESS regression. **c**. Gene set enrichment analysis (one-sided Fisher’s exact test) using the top 200 genes from each neurodevelopmental signature with significantly up-regulated (adjusted P-value < 0.05) t-hCO genes from pseudobulk GluN clusters (neurogenesis, neuronal differentiation, and synaptogenesis signatures), Astroglia clusters (astrogenesis signature), and from pseudobulk of all clusters (myelination-related signature). Line denotes Bonferroni corrected P-value of 0.05. **d**. Gene expression (pseudobulk and scaled) of the top 50 significantly upregulated t-hCO genes (ranked by differential expression p-value significance) for each signature. **e–f**. Heat maps of t-hCO (**e**) and hCO (**f**) cluster overlap by RNA-seq integration with primary human fetal cortical cell clusters^19,20^. Cell cluster labels are from original studies. RG, radial glia; Cyc.prog, cycling progenitors; tRG, truncated radial glia, mGPC, multipotent glial progenitor cell; OPC/Oligo, oligodendrocyte progenitor cell/oligodendrocyte; nIPC, neuronal intermediate progenitor cell; GluN, glutamatergic neuron; CGE IN, caudal ganglionic eminence interneuron; MGE IN, medial ganglionic eminence interneuron; EC, endothelial cell; MG, microglia; Peric., Pericytes; PgG2M and PgS, cycling progenitors; IP, intermediate progenitor; oRG, outer radial glia; End, endothelial cell; Per, pericyte; vRG, ventricular radial glia; ExM, maturing excitatory neuron; ExN, excitatory neuron; ExM.U, maturing upper layer excitatory neuron; ExDp, excitatory deep layer neuron; In, interneuron. **g–h**. UMAP visualization of GluN cell type classification of t-hCO (**g**) and hCO (**h**) using label transfer (methods) from adult human cortical single nuclei RNA-seq reference datasets. Transfer labels from dissected cortical layers of medial temporal gyrus (MTG)^21^ shown in **g** and to the left in **h**. Right (**h**): transfer labels from annotated GluN subclasses from motor cortex (M1)^22^. **I–j**. Heat maps of t-hCO (**i**) and hCO (**j**) cluster overlap by RNA-seq integration with primary human adult cortical cell clusters^21,22^. Cell cluster labels are from original studies. Exc, excitatory neuron; Inh, inhibitory neuron; Astro, astrocyte; Endo, endothelial cells; CT, corticothalamic cell; ET, extratelencephalic cell; IT, intratelencephalic cell; micro, microglia; NP, near-projecting; oligo, oligodendrocyte; OPC, oligodendrocyte precursor; PVM, perivascular macrophage; VLMC, vascular and leptomeningeal cells.

**Extended Data Fig. 6:**
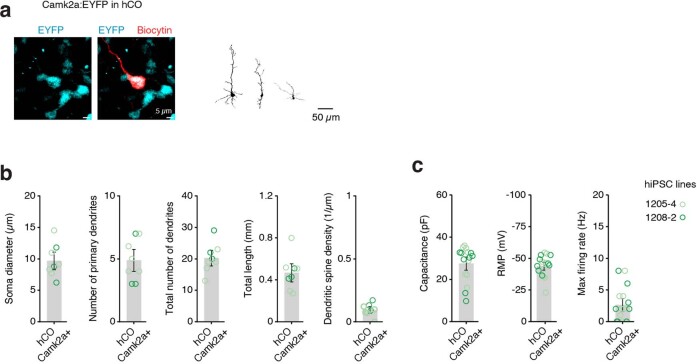
Electrophysiological and morphological properties of glutamatergic hCO neurons. **a**. Example 3D-reconstruction of biocytin-filled Camk2α:eYFP-expressing hCO neurons at 8 months. **b**. Morphological properties of 3D reconstructed Camk2α^+^ hCO neurons (n = 8). **c**. Electrophysiological properties of Camk2α^+^ hCO neurons (n = 16 neurons). Data are presented as mean ± SEM.

**Extended Data Fig. 7:**
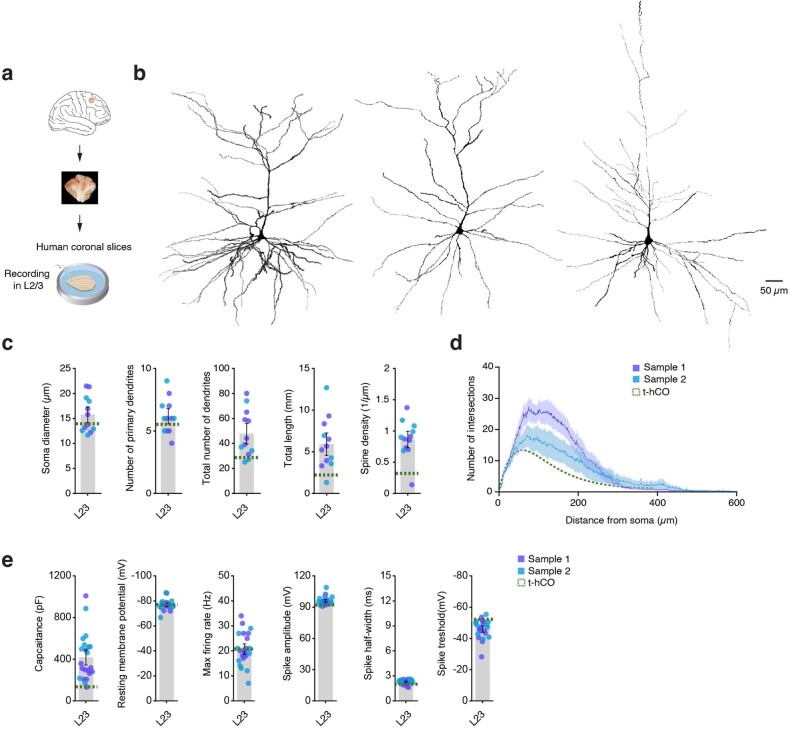
Electrophysiological and morphological properties of cortical neurons from postnatal human cerebral cortex. **a**. Schematics showing the location of resected specimens and recording conditions. **b**. 3D-reconstruction of biocytin-filled human L2/3 pyramidal neurons. **c**. Quantification of the soma diameter, number of primary branches, total number of dendrites, total length and spine density (n = 12 L2/3 neurons from 2 specimens). **d**. Sholl analysis comparison of the dendritic complexity of L2/3 neurons (n = 7 L2/3 neurons from sample 1 and n = 8 neurons from sample 2). **e**. Quantification of membrane capacitance, resting membrane potential, maximal firing rate, spike amplitude, spike half-width and spike threshold of L2/3 pyramidal neurons (n = 22 L2/3 neurons from 2 specimens). Dotted lines show t-hCO for comparison. Data are presented as mean ± SEM.

To further assess how maturation of t-hCO relates to the developing human brain, we performed transcriptomic comparisons with human fetal^19,20^ and adult^21,22^ cortical cell types, as well as with developing bulk cortical gene expression data^23^ (Extended Data Fig. 5). Consistent with previous work^24^, the global transcriptomic maturation state of both hCO and t-hCO at 7–8 months of differentiation broadly matched in vivo developmental timing and was most equivalent to the late fetal period (Extended Data Fig. 5a). Of note, we observed increased transcriptomic maturation of t-hCO compared with age-matched hCO, as well as upregulation of transcripts associated with synaptogenesis, astrogenesis and myelination (Extended Data Fig. 5b–d). At the cell-class level, we found evidence for more refined cortical layer subtypes in t-hCO, with glutamatergic neuron cluster overlap to adult L2/3, L5 and L6 neuronal subclasses (Fig. 1i). By contrast, there was more limited cluster overlap between t-hCO glutamatergic neurons and fetal cortical neurons from the second trimester **(**Extended Data Fig. 5e–j). To determine whether t-hCO neurons functionally resemble postnatal human neocortical neurons, we performed electrophysiological recordings and anatomical reconstructions of human cortical L2/3 pyramidal neurons in acute slices from postnatal human cortex (Extended Data Fig. 7a). The electrophysiological properties of L2/3 pyramidal neurons were similar to those of t-hCO pyramidal neurons (Extended Data Fig. 7e). Morphologically, L2/3 neurons from postnatal human samples were much more similar to t-hCO than to hCO, although L2/3 cells were longer, contained more branches overall and had higher spine density (Fig. 3g and Extended Data Fig. 7b–d).

**Extended Data Fig. 8:**
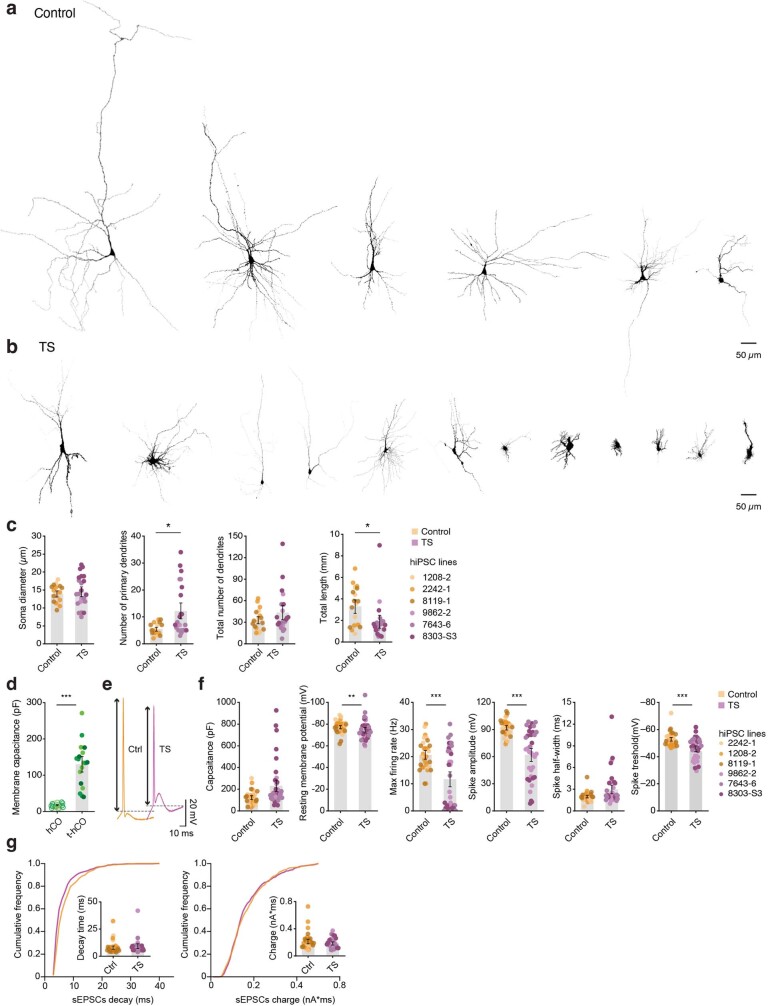
Morphological and electrophysiological properties of control and TS t-hCO neurons. **a–c**. Morphological properties of control and TS t-hCO neurons. **a–b**. Examples of 3D-reconstructed t-hCO neurons derived from control Line identities of filled cells from left to right, Control: 1208-2; 2242-1; 8119-1; 2242-1; 2242-1; 1208-2; and TS: 8303-S3; 8303-S3; 7643-6; 7643-6; 9862-2; 8303-S3; 8303-S3; 8303-S3; 8303-S3; 9862-2; 8303-S3; 7643-6. **c**. Quantification of the soma diameter, number of primary branches, total number of dendrites and total length (control: n = 19 neurons, TS: n = 23 neurons, P = 0.592, *P = 0.025; control: n = 19 neurons, TS: n = 21 neurons P = 0.7627, * P = 0.0112). **d**. Comparison of membrane capacitance in hCO and t-hCO (hCO: n = 25 neurons, t-hCO: n = 18 neurons, ***P < 0.0001). **e–g**. Electrophysiological properties of control and TS t-hCO neurons. **e**. Example traces of a single AP firing in control and TS t-hCO neurons, showing differences in AP height (black arrows) and threshold (gray dashed line). **f**. Comparison of electrophysiological properties in control and TS t-hCO neurons; membrane capacitance (Control: n = 30 neurons, TS: n = 36 neurons, P = 0.0848), resting membrane potential (Control: n = 31 neurons, TS: n = 33 neurons, **P < 0.0044), maximal firing rate (Control: n = 29 neurons, TS: n = 36 neurons, ***P < 0.0001), spike amplitude (Control: n = 31 neurons, TS: n = 35 neurons, ***P < 0.0001), spike half-width (Control: n = 31 neurons, TS: n = 34 neurons, P = 0.0984) and spike threshold (Control: n = 31 neurons, TS: n = 34 neurons, ***P < 0.0001). **g**. Cumulative frequency plots and quantification of spontaneous EPSCs decay time and charge (Control: n = 32 neurons; TS: n = 26 neurons, P = 0.0744, P = 0.4812). Data are presented as mean ± SEM.

The ability of t-hCO to recapitulate advanced morphological and functional features in human cortical neurons prompted us to verify whether t-hCO can be used to uncover disease phenotypes. We focused on TS, a severe neurodevelopmental disease, caused by a gain-of-function mutation in the gene encoding Ca_V_1.2, which initiates activity-dependent gene transcription in neurons^14^. We generated hCO from three patients with TS that carry the most common substitution (p.G406R) and from three control participants (Fig. 3a). Following transplantation, we found that TS neurons had an altered dendritic morphology compared with controls (Fig. 3b and Extended Data Fig. 8a,b), with a twofold increase in the number of primary dendrites and an overall reduction in mean and total dendrite length (Fig. 3c and Extended Data Fig. 8c). This was associated with an increase in synaptic spine density and higher frequency of spontaneous EPSCs in TS compared with control neurons (Fig. 3d–f and Extended Data Fig. 8g). Further analysis revealed an abnormal dendritic branching pattern in TS t-hCO versus control, but not in TS hCO in vitro at a similar differentiation stage (Fig. 3g). This is consistent with an activity-dependent dendritic retraction in TS that we previously reported^25^ and highlights the ability of this transplantation platform to reveal disease phenotypes in an in vivo context.

**Fig. 4:**
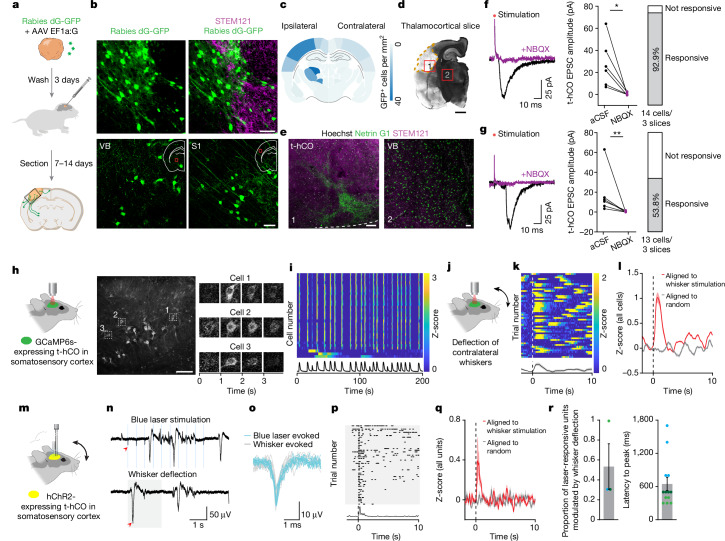
Transplanted hCO receive sensory-related inputs. **a**, Schematic of the rabies-tracing experiment. **b**, GFP and human-specific STEM121 expression between t-hCO and the rat cortex (top). GFP expression in the rat ipsilateral ventrobasal (VB) nucleus (bottom left) and the ipsilateral S1 (bottom right) is also shown. Scale bars, 50 μm. Red squares indicate the region of the brain from which the image is taken. **c**, Quantification of GFP-expressing cells (*n* = 4 rats). **d**,**e**, Netrin G1^+^ thalamic terminals in t-hCO. In **d**, a coronal section containing t-hCO and the VB nucleus is shown. Scale bar, 2 mm. In **e**, Netrin G1 expression and STEM121 expression in t-hCO (left) and VB neurons (right) are shown. Scale bars, 50 µm. Orange dashed line indicates border of t-hCO. **f**,**g**, Current traces from t-hCO neurons following electrical stimulation in the rat S1 (**f**) or the internal capsule (**g**), with (purple) or without (black) NBQX (left). EPSC amplitude with or without NBQX (*n* = 6 S1 neurons, *P = 0.0119; and *n* = 6 internal capsule neurons, **P = 0.0022) (middle). Percentage of t-hCO neurons that displayed EPSCs in response to electrical stimulation of the rat S1 (**f**) or internal capsule (**g**) (right). aCSF, artificial cerebrospinal fluid. **h**, Schematic of the 2P-imaging experiment (left). GCaMP6s expression in t-hCO (middle). Scale bar, 100 μm. Timelapse of GCaMP6s fluorescence (right). **i**, Z-scored fluorescence of spontaneous activity. **j**, Schematic of whisker stimulation. **k**, Single-trial z-scored 2P fluorescence traces aligned to whisker deflection at time zero (dashed line) in an example cell. **l**, Population-averaged z-scored responses of all cells aligned to whisker deflection at time zero (dashed line) (red) or randomly generated timestamps (grey). **m**, Schematic of the optotagging experiment. **n**, Raw voltage traces from an example t-hCO unit during blue laser stimulation or whisker deflection. The red arrowheads indicate the first light-evoked (top) or whisker deflection-evoked (bottom) spike. Grey shading indicates period of whisker deflection. **o**, Spike waveforms of light and whisker deflection responses. **p**, Single-trial spiking aligned to whisker deflection in an example cell. 0 indicates whisker deflection (dashed line). **q**, Population-averaged z-scored firing rates of all light-responsive units aligned to whisker deflection at time zero (dashed line) (red) or randomly generated timestamps (grey). **r**, Proportion of light-responsive units significantly modulated by whisker deflection (*n* = 3 rats) (left). Latency to peak z-score (*n* = 3 rats; *n* = 5 (light green), *n* = 4 (dark green) and *n* = 4 (cyan) whisker deflection-modulated units per rat) (right). Data are presented as mean ± s.e.m.

**Extended Data Fig. 9:**
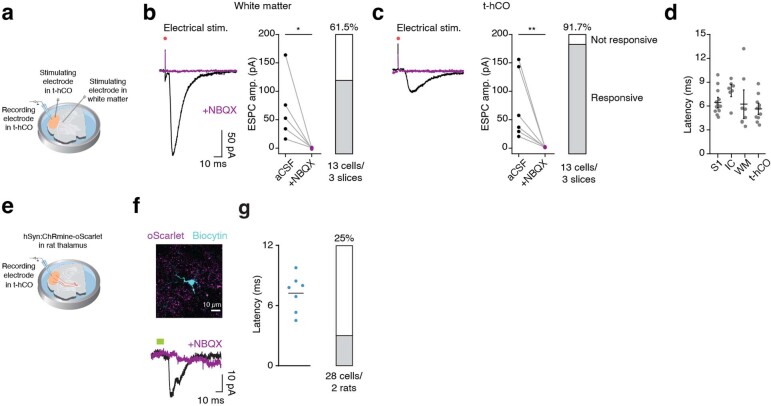
Electrophysiological characterization of inputs onto t-hCO. **a**. Schematic of experimental preparation for electrically activating rat tissue while performing whole cell recordings from t-hCO neurons. **b**. *Left*, current traces from a representative t-hCO neuron following electrical stimulation in white matter with (purple) or without (black) NBQX. *Middle*, quantification of EPSC amplitude with or without NBQX (paired t-test, *P = 0.0295). *Right*, percentage of t-hCO neurons that displayed EPSCs in response to electrical stimulation in white matter. **c**. *Left*, Current traces from a representative t-hCO neuron following electrical stimulation of t-hCO with (purple) or without (black) NBQX. *Middle*, quantification of EPSC amplitude with or without NBQX (Wilcoxon test, **P = 0.0022). *Right*, percentage of t-hCO neurons that displayed EPSCs in response to electrical stimulation of t-hCO. **d**. Latency to EPSC in t-hCO neurons following electrical stimulation of somatosensory cortex (S1, n = 13 neurons), internal capsule (IC, n = 7 neurons), white matter (WM, n = 8 neurons), or t-hCO (n = 11 neurons). **e**. Schematic of experimental preparation for optogenetically activating rat thalamic terminals in t-hCO while performing whole cell recordings from t-hCO neurons. **f**. *Top*, example recorded t-hCO neuron. *Bottom*, current traces from a representative t-hCO neuron following optogenetic activation of rat thalamic terminals in t-hCO with (purple) or without (black) NBQX. **g**, *Left*, latency to EPSC in t-hCO neurons following optogenetic activation of rat thalamic terminals in t-hCO (n = 7 neurons, 2 animals). *Right*, percentage of t-hCO neurons that displayed EPSCs in response to optogenetic activation of rat thalamic terminals in t-hCO. Data are presented as mean ± SEM.

We next asked to what extent t-hCO cells functionally integrate into the rat S1. The S1 in rodents receives robust synaptic input from the ipsilateral ventrobasal nucleus and the posterior nucleus of the thalamus, as well as from the ipsilateral motor and secondary somatosensory cortices and contralateral S1 (Fig. 4a). To reconstruct innervation patterns, we infected hCO with rabies-dG-GFP/AAV-G and, after 3 days, hCO were transplanted into the rat S1. At 7–14 days post-transplantation, we observed dense GFP expression in neurons in the ipsilateral S1 and ventrobasal nucleus (Fig. 4b,c). In addition, antibody staining for the thalamic marker netrin G1, revealed the presence of thalamic terminals in t-hCO (Fig. 4d,e). To assess whether these afferent projections were capable of evoking synaptic responses in t-hCO cells, we performed whole-cell recordings from human cells in acute thalamocortical slices^26^. Electrical stimulation of the nearby fibres in the rat S1, internal capsule, white matter, t-hCO or optogenetic activation of opsin-expressing thalamic terminals in t-hCO evoked short-latency EPSCs in t-hCO neurons, which were blocked by application of the AMPA receptor antagonist NBQX (Fig. 4f,g and Extended Data Fig. 9a–g). These data demonstrate that t-hCO become anatomically integrated into the rat brain, and are capable of being activated by host rat tissue.

**Extended Data Fig. 10:**
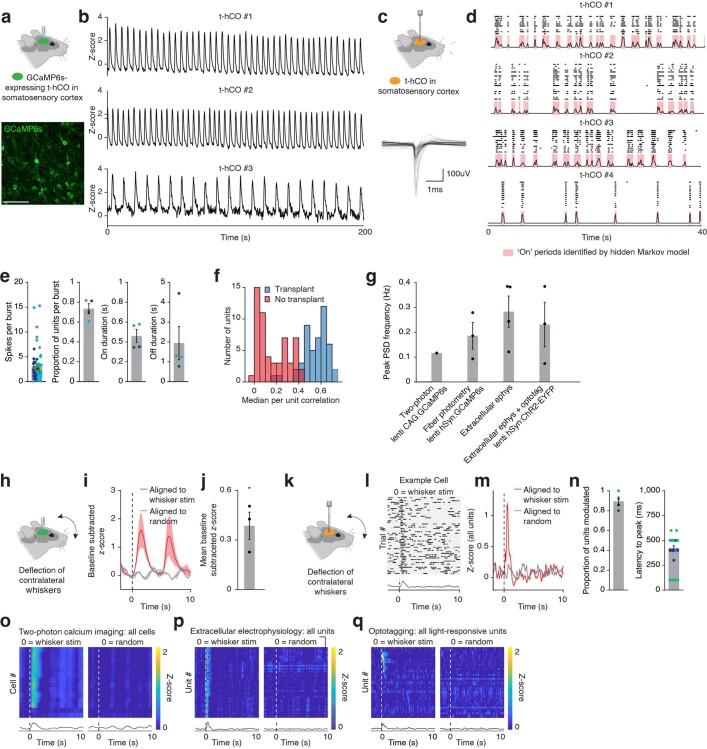
Characterization of t-hCO activity *in vivo*. **a–g**. Characterization of spontaneous activity in t-hCO *in vivo*. **a–c**. Fiber photometry recordings of spontaneous t-hCO activity *in vivo*. **a**. *Top*, Schematic of experimental preparation. *Bottom*, representative image of GcaMP6s expression in t-hCO. Scale bar, 100 μm. **b**. Example z-scored fluorescence traces from awake recording of spontaneous activity. **c–f**. Extracellular recordings of spontaneous t-hCO activity *in vivo*. **c**. *Top*, Schematic of experimental preparation. *Bottom*, average waveforms of putative t-hCO units. **d**. Hidden Markov model used to identify ‘on’ and ‘off’ periods of population activity. *Top*, raster plot of spontaneous activity of simultaneously recorded units. *Bottom*, population averaged z-scored activity with ‘on’ states identified by Hidden Markov model overlaid in red. **e**. Quantification of spontaneous spiking activity. *Left*, number of spikes each unit contributed to each burst (n = 4 rats, 10 (dark green), 12 (light green), 19 (cyan), and 14 (blue) units per rat). *Left center*, proportion of recorded units that were engaged in each burst (n = 4 rats). *Right center*, ON period duration (n = 4 rats). *Right*, OFF period duration (n = 4 rats). **f**. Histogram of median correlations of each unit with all other simultaneously recorded units recorded in transplanted rats (putative human units, blue, n = 48 units from 4 rats) or non-transplanted rats (rat units, red, n = 56 units from 3 rats, P < 0.0001). **g**. Peak power spectral density frequency of spontaneous activity across all recording modalities used (two photon n = 1 rat, fiber photometry n = 3 rats, extracellular electrophysiology n = 4 rats, optotag n = 3 rats). **h–q**. Characterization of evoked activity in t-hCO *in vivo*. **h–j**. Fiber photometry recordings of t-hCO activity in response to whisker deflection. **h**. Schematic of experimental preparation for fiber photometry recording of t-hCO neurons in response to whisker deflection. **i**. Z-scored responses to whisker deflection at time zero (red) or randomly generated timestamps (grey) averaged across animals (n = 3 rats). **j**. Quantification of mean z-score following whisker stimulation compared to baseline (n = 3 rats, *P = 0.0430). **k–n**. Extracellular electrophysiological recordings of t-hCO activity in response to whisker deflection. **k**. Schematic of experimental preparation for extracellular electrophysiological recordings of t-hCO activity in response to whisker deflection. **l**. Single trial responses to whisker stimulation from a representative example single unit. *Top*, raster plot of single trial spiking activity aligned to whisker deflection. *Bottom*, trial-averaged z-scored firing rate. **m**. Population-averaged z-scored firing rates of all cells aligned to whisker deflection at time zero (red) or randomly generated timestamps (grey). **n**. Quantification of evoked spiking activity. *Left*, proportion of units significantly modulated by whisker deflection (n = 4 rats). *Right*, latency to peak z-score (n = 4 rats, n = 8 (dark green), 11 (light green), 12 (cyan), and 11 (blue) whisker deflection-modulated units per rat). **o–q**. Heatmaps of trial-averaged responses of all identified putative t-hCO cells across different recording modalities. **o**. *Top*, heatmap of trial-averaged z-scored fluorescence traces from all t-hCO cells recorded with two-photon calcium imaging aligned to whisker deflection (left) or randomly generated timestamps (right). *Bottom*, population-averaged z-scored fluorescence traces (n = 14 cells from 1 rat). **p**. *Top*, heatmap of trial-averaged z-scored firing rates from all t-hCO units recorded with extracellular electrophysiology aligned to whisker deflection (left) or randomly generated timestamps (right). *Bottom*, population-averaged z-scored firing rates (n = 42 units from 4 rats). **q**. *Top*, heatmap of trial-averaged z-scored firing rates from all opto-tagged t-hCO units recorded with extracellular electrophysiology aligned to whisker deflection (left) or randomly generated timestamps (right). *Bottom*, population-averaged z-scored firing rates (n = 31 units from 3 rats). Data are presented as mean ± SEM.

We next asked whether t-hCO could be activated by sensory stimuli within an in vivo context. We transplanted hCO expressing the genetically encoded calcium indicator GCaMP6s into the rat S1. After 150 days, we conducted fibre photometry or two-photon calcium imaging (Fig. 4h and Extended Data Fig. 10a). We found that t-hCO cells exhibited synchronous, rhythmic activity (Fig. 4i, Extended Data Fig. 10b and Supplementary Video 1). To characterize the spiking activity of t-hCO, we performed extracellular electrophysiological recordings in anaesthetized, transplanted rats (Extended Data Fig. 10c–f). We generated stereotactic coordinates based on images acquired from MRI; these recorded units thus represent putative human neurons, although electrophysiology alone does not permit species-of-origin identification. We observed synchronous bursts of activity (Extended Data Fig. 10d). Spiking bursts were approximately 460 ms in length and were separated by approximately 2-s-long silent periods (Extended Data Fig. 10d,e). Individual units fired on average approximately three spikes per burst, and each burst recruited approximately 73% of recorded units. The activity of individual units was highly correlated, and these correlations were higher than those observed in units identified in non-transplanted animals recorded under the same conditions (Extended Data Fig. 10f). To further characterize the spiking responses of neurons of definitive human origin, we performed optotagging experiments in anaesthetized rats transplanted with hCO expressing the light-sensitive cation channel channelrhodopsin-2 (hChR2), in which t-hCO neurons were identified by their short-latency (less than 10 ms) response to blue light stimulation (Fig. 4m–o). t-hCO neurons displayed spontaneous bursts of activity of similar frequency to those observed with calcium imaging, as well as to electrophysiological recordings performed within t-hCO without optotagging (Extended Data Fig. 10c–g). Spontaneous activity was not observed in stage-matched hCO recorded in vitro. To assess whether t-hCO could be activated by sensory stimuli, we briefly deflected the rat whiskers contralateral to t-hCO (Fig. 4j,m and Extended Data Fig. 10h,k). In accordance with previous studies^8,10^, a subset of t-hCO cells displayed increases in activity in response to whisker deflection that were not observed when data were aligned to randomized timestamps (Fig. 4k–q and Extended Data Fig. 10h–q). Indeed, approximately 54% of optotagged single units displayed significant increases in firing rates following whisker stimulation that peaked after approximately 650 ms (Fig. 4r). Together, these data suggest that t-hCO receive appropriate functional inputs and can be activated by environmental stimuli.

**Fig. 5:**
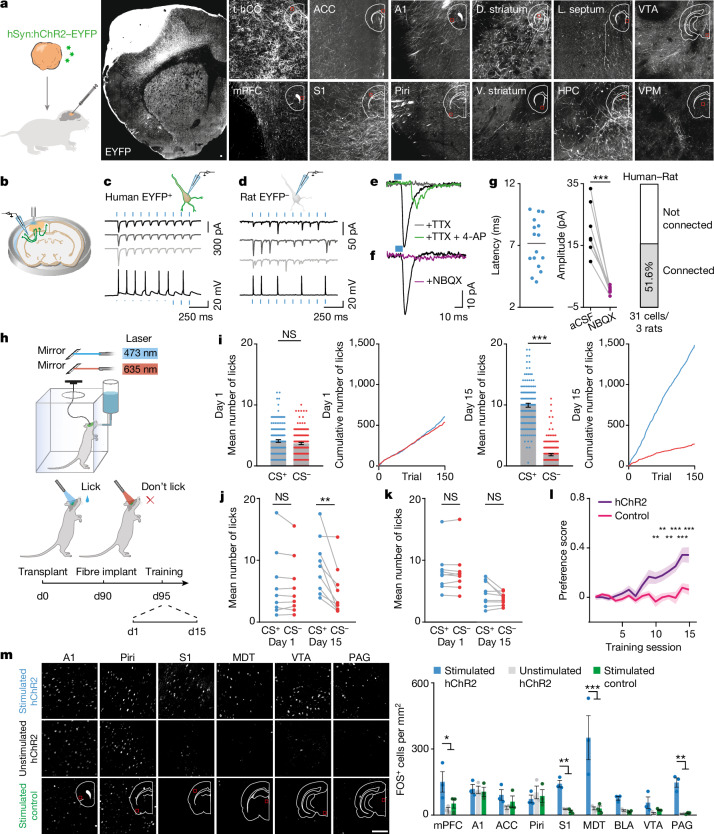
Transplanted hCO make functional connections onto rat neurons and modulate behaviour. **a**, Schematic of axon tracing (left). t-hCO EYFP expression (right). Scale bar, 100 μm. A1, auditory cortex; ACC, anterior cingulate cortex; d. striatum, dorsal striatum; HPC, hippocampus; l. septum, lateral septum; mPFC, medial prefrontal cortex; piri, piriform cortex; v. striatum, ventral striatum; VPM, ventral posteromedial nucleus of thalamus; VTA, ventral tegmental area. Red squares indicate the region of the brain from which the image is taken. **b**, Schematic of the stimulation experiment. **c**,**d**, Example blue light-evoked photocurrents (top) and voltage responses (bottom) in human EYFP^+^ t-hCO (**c**) or rat EYFP^–^ cells (**d**). **e**,**f**, Current traces from rat neurons following blue light stimulation of t-hCO axons with TTX and 4-AP (green), TTX (grey) or in aCSF (black) (**e**), or with (purple) or without (black) NBQX (**f**). **g**, Latency of blue light-evoked responses in rat cells (*n* = 16 cells); horizontal bar indicates mean latency (7.13 ms) (left). Amplitude of light-evoked EPSCs recorded with or without NBQX (*n* = 7 cells; ***P < 0.0001) (middle). Percentage of rat cells that demonstrated EPSCs in response to blue light (right). **h**, Schematic of the behavioural task. d0, day 0. **i**, Performance of an example animal on day 1 (left) or day 15 (right) of training. The mean number of licks performed on day 1 (left) or day 15 (right centre) (*n* = 150 blue light trials, *n* = 150 red light trials; ***P < 0.0001). Cumulative lick count across red and blue light trials on day 1 (left centre) or day 15 (right). NS, not significant. **j**,**k**, Behavioural performance of all animals transplanted with t-hCO expressing hChR2–EYFP (**j**) or control fluorophore (**k**) on day 1 or day 15 (hChR2–EYFP: *n* = 9 rats, **P = 0.0049; control: *n* = 9, *P* = 0.1497). **l**, Evolution of preference score (*n* = 9 hChR2, *n* = 9 control; **P < 0.001, ***P < 0.0001). **m**, FOS expression in response to optogenetic activation of t-hCO in the S1. Images of FOS expression (left), and quantification (*n* = 3 per group; *P < 0.05, **P < 0.01 and ***P < 0.001) (right) are shown. Scale bar, 100 μm. Data are presented as mean ± s.e.m. BLA, basolateral amygdala; MDT, mediodorsal nucleus of the thalamus; PAG, periaqueductal grey.

We next investigated whether t-hCO can engage rat circuits to drive behaviour. We first examined whether t-hCO neurons send axonal projections into surrounding rat tissue. We infected hCO with a lentivirus encoding hChR2 fused to EYFP (hChR2–EYFP). We observed EYFP expression in ipsilateral cortical regions including auditory, motor and somatosensory cortices, as well as in subcortical regions including the striatum, hippocampus and thalamus, 110 days later (Fig. 5a). To assess whether these efferent projections were capable of evoking synaptic responses in rat cells, we optically activated hChR2–EYFP-expressing t-hCO cells while recording from cortical rat cells in acute brain slices. Activation of t-hCO axons with blue light evoked short-latency EPSCs in rat pyramidal cortical neurons, which were blocked by NBQX (Fig. 5b–g). Moreover, these responses could be blocked by application of tetrodotoxin (TTX) and recovered by 4-amino-pyridine (4-AP), indicating that they were evoked by monosynaptic connections^27^ (Fig. 5e).

We finally asked whether t-hCO were capable of modulating rat behaviour. To test this, we transplanted hCO expressing hChR2–EYFP into the S1, and 90 days later, we implanted an optical fibre into t-hCO for light delivery. We then trained rats on a modified operant conditioning paradigm (Fig. 5h). We placed animals into a behavioural testing chamber and applied randomly interleaved presentations of 5-s-long blue (473 nm) and red (635 nm) laser stimulations. Animals received a water reward if they licked during the blue light stimulation, but not if they licked during the red light stimulation. On the first day of training, animals showed no difference in their licking behaviour during either blue or red light stimulation. However, on day 15, animals transplanted with hCO expressing hChR2–EYFP showed increased licking during blue light stimulation compared with red light stimulation. These changes in licking behaviour were not observed in control animals transplanted with hCO expressing a control fluorophore (learning success rate: hChR2 89%, EYFP 0%, Fig. 5i–l and Supplementary Video 2). These data suggest that t-hCO cells can activate rat neurons to drive reward-seeking behaviours. To explore what rat neural circuits might be engaged by t-hCO to drive these changes in behaviour, we optogenetically activated t-hCO in trained animals and collected tissue after 90 min. Immunohistochemistry revealed expression of the activity-dependent protein FOS in several brain regions implicated in motivated behaviours, including the medial prefrontal cortex, the mediodorsal thalamus and the periaqueductal grey, and this was not observed in unstimulated control animals or in stimulated control animals that did not express an opsin (Fig. 5m). Together, these data demonstrate that t-hCO can modulate the activity of rat neurons to drive behaviour.

## Discussion

Neural organoids represent a promising system to explore human development and disease in vitro, but they are limited by the lack of circuit connectivity that exists in vivo. We developed a novel platform in which we transplanted hCO into the S1 of early-postnatal immunocompromised rats to examine human cell development and function in vivo. We have demonstrated that t-hCO develop mature cell types^28^ that are not seen in vitro, and that t-hCO integrate both anatomically and functionally into the rodent brain. Integration of t-hCO into rodent neural circuits allowed us to establish links between the activity of human cells and learned animal behaviour, showing that t-hCO neurons can modulate rat neuron activity to drive behavioural responses.

The platform that we have described has several advantages in comparison to previous studies that have transplanted human cells into the rodent brain^7–12^. First, we transplanted hCO into the developing cerebral cortex of early-postnatal rats, which probably favours anatomical and functional integration. Second, MRI monitoring of t-hCO allowed us to examine graft position and growth in living animals, enabling us to perform long-term studies across multiple animals and establish reliability across multiple hiPS cell lines. Finally, we engrafted intact organoids, rather than a dissociated single-cell suspension, which is less disruptive to human cells and probably facilitates integration and the generation of a unit of human cortical neural cells in the rat brain.

We recognize that despite the advances that this platform offers, there are temporospatial and cross-species limitations that preclude the formation of human neural circuits with high fidelity even after transplantation at early stages in development. For example, it is currently unclear whether the spontaneous activity observed in t-hCO represents a developmental phenotype, similar to rhythmic activity observed during cortical development^29^, or is related to the lack of inhibitory cell types present in t-hCO. Similarly, it is uncertain to what extent the lack of lamination in t-hCO might influence circuit connectivity^30^. Future work will be aimed at incorporating other cell types such as human microglia, human endothelial cells and various proportions of GABAergic interneurons, as has been shown in vitro using assembloids^6^, as well as understanding how neural integration and processing might be altered in patient-derived t-hCO at the transcriptional, synaptic and behavioural levels.

Overall, this in vivo platform represents a powerful resource to complement in vitro studies of human brain development and disease. We anticipate that this platform will allow us to uncover new circuit-level phenotypes in patient-derived cells that have otherwise been elusive and to test novel therapeutic strategies.

## Methods

### hCO generation

We generated hCO from hiPS cells as previously described^2,5^. To initiate the generation of hCO from hiPS cells cultured on feeders, intact hiPS cell colonies were lifted from the plates using dispase (0.35 mg ml^−1^) and transferred to ultra-low attachment plastic dishes (Corning) in hiPS cell medium supplemented with the two SMAD inhibitors dorsomorphin (5 μM; P5499, Sigma-Aldrich) and SB-431542 (10 μM; 1614, Tocris) and the ROCK inhibitor Y-27632 (10 μM; S1049, Selleckchem). For the first 5 days, the hiPS cell medium was changed every day and supplemented with dorsomorphin and SB-431542. On the sixth day in suspension, neural spheroids were transferred to neural medium containing neurobasal-A (10888, Life Technologies), B-27 supplement without vitamin A (12587, Life Technologies), GlutaMax (1:100, Life Technologies), penicillin and streptomycin (1:100, Life Technologies) and supplemented with the epidermal growth factor (EGF; 20 ng ml^−1^; R&D Systems) and fibroblast growth factor 2 (FGF2; 20 ng ml^−1^; R&D Systems) until day 24. From day 25 to day 42, the medium was supplemented with brain-derived neurotrophic factor (BDNF; 20 ng ml^−1^, Peprotech) and neurotrophin 3 (NT3; 20 ng ml^−1^; Peprotech) with medium changes every other day. From day 43 onward, hCO were maintained in unsupplemented neurobasal-A medium (NM; 1088022, Thermo Fisher) with medium changes every 4–6 days. For the generation of hCO from hiPS cells cultured on feeder-free conditions, hiPS cells were incubated with accutase (AT-104, Innovate Cell Technologies) at 37 °C for 7 min, dissociated into single cells and seeded into AggreWell 800 plates (34815, STEMCELL Technologies) at a density of 3 × 10^6^ single cells per well in Essential 8 medium supplemented with the ROCK inhibitor Y-27632 (10 μM; S1049, Selleckchem). After 24 h, spheroids were collected from each microwell by pipetting medium in the well up and down and transferring it into ultra-low attachment plastic dishes (3262, Corning) containing Essential 6 medium (A1516401, Life Technologies) supplemented with dorsomorphin (2.5 μM; P5499, Sigma-Aldrich) and SB-431542 (10 μM; 1614, Tocris). From day 2 to day 6, Essential 6 medium was changed every day and supplemented with dorsomorphin and SB-431542. From the sixth day in suspension, neural spheroids were transferred to neurobasal medium and maintained as described above.

### Transplantation into athymic newborn rats

All animal procedures followed animal care guidelines approved by Stanford University’s Administrative Panel on Laboratory Animal Care (APLAC). Pregnant RNU euthymic (rnu/+) rats were purchased (Charles River Laboratories) or bred in house. Animals were maintained under a 12-h light–dark cycle and provided food and water ad libitum. Three-to-seven-day-old athymic (*FOXN1*^–^^/^^–^) rat pups were identified by immature whisker growth before culling. Pups (male and female) were anaesthetized with 2–3% isoflurane and mounted on a stereotaxic frame. A craniotomy, about 2–3 mm in diameter, was performed above the S1, preserving the dura intact. Next, the dura mater was punctured using a 30-G needle (approximately 0.3 mm) close to the lateral side of the craniotomy. A hCO was next moved onto a thin 3 × 3-cm parafilm and excess medium was removed. Using a Hamilton syringe connected to a 23 G, 45° needle, the hCO was gently pulled into the most distal tip of the needle. The syringe was next mounted on a syringe pump connected to the stereotaxic device. The sharp tip of the needle was next positioned above the 0.3-mm-wide pre-made puncture in the dura mater (*z* = 0 mm), the syringe was reduced 1–2 mm (*z* = approximately –1.5 mm), and until a tight seal between the needle and the dura mater was formed. Next, the syringe was elevated to the centre of the cortical surface at *z* = –0.5 mm, and the hCO was ejected at a speed of 1–2 µl per minute. After injection of hCO was completed, the needle was retracted at a rate of 0.2–0.5 mm per minute, the skin was closed, and the pups were immediately placed on a warmed heat pad until complete recovery. Some animals were engrafted bilaterally.

### MRI of transplanted rats

All animal procedures followed animal care guidelines approved by Stanford University’s APLAC. Rats (more than 60 days post-transplantation) were anaesthetized with 5% isoflurane for induction and 1–3% isoflurane during imaging. For imaging, an actively shielded Bruker 7 Tesla horizontal bore scanner (Bruker Corp.) with International Electric Company (IECO) gradient drivers, a 120-mm inner diameter shielded gradient insert (600 mT/m, 1,000 T/m/s), AVANCE III electronics, eight-channel multi-coil radiofrequency and multinuclear capabilities, and the supporting Paravision 6.0.1 platform were used. Acquisitions were performed with an 86 mm inner diameter actively de-couplable volume radiofrequency coil with a four-channel cryo-cooled receive-only radiofrequency coil. Axial 2D Turbo-RARE (repetition time = 2,500 ms, echo time = 33 ms, 2 averages) 16 slice acquisitions were performed with 0.6–0.8-mm slice thickness, with 256 × 256 samples. Signal was received with a 2-cm inner diameter quadrature transmit–receive volume radiofrequency coil (Rapid MR International, LLC). Finally, 3D volume rendering and analysis were performed using Imaris (BitPlane) built-in surface estimation functions. Successful transplantations were defined as transplantations that resulted in a continuous area of T2-weighted MRI signal in the transplanted hemisphere. Failed transplantations were defined as transplantations that did not result in a continuous area of T2-weighted MRI signal in the transplanted hemisphere. Subcortical t-hCO were excluded from subsequent analyses.

### Lentivirus labelling and G-deleted rabies infections

To stably express GCaMP6s in hCO for two-photon calcium imaging, hiPS cells were infected with pLV[Exp]-EF1a::GcaMP6s-WPRE-Puro followed by antibiotic selection. In brief, the cells were dissociated with EDTA and suspended at a density of approximately 300,000 cells in 1 ml in Essential 8 medium in the presence of polybrene (5 µg ml^−1^) and 15 µl of virus. Cells were then incubated in suspension for 60 min and plated at a density of 50,000 cells per well. Once confluent, cells were treated with 5–10 µg ml^−1^ puromycin for 5–10 days or until stable colonies appeared. Acute infections of hCO were performed as previously described^5^ with a few modifications. In brief, day 30–45 hCO were transferred to 1.5-ml microcentrifuge Eppendorf tubes containing 100 µl neural medium. Next, approximately 90 µl of medium was removed and 3–6 µl of high-titre lentivirus (0.5 × 10^8^ to 1.2 × 10^9^) was added to the tube, and the hCO were moved to the incubator for 30 min. Next, 90–100 µl medium was added to each tube and the tubes were returned to the incubator overnight. The next day, hCO were transferred to fresh neural medium in low-attachment plates. After 7 days, hCO were moved to glass-bottom 24-well plates for imaging and infection quality assessment. pLV[Exp]-SYN1::EYFP-WPRE and pLV[Exp]-SYN1::hChR2-EYFP-WPRE were generated by VectorBuilder. Lentivirus was used for most experiments as it is incorporated into the host genome permitting reporter expression in the infected cell lineage. For rabies tracing, day 30–45 hCO were co-infected with rabies-ΔG-eGFP and AAV-DJ-EF1a-CVS-G-WPRE-pGHpA (plasmid #67528, Addgene), thoroughly washed over the course of 3 days, transplanted into the rat S1 and maintained in vivo for 7–14 days.

### Tissue preparation, immunohistochemistry and quantification

For immunocytochemistry, animals were anaesthetized and transcardially perfused with PBS followed by 4% paraformaldehyde (PFA in PBS; Electron Microscopy Sciences). Brains were post-fixed with 4% PFA for either 2 h or overnight at 4 °C, cryopreserved in 30% sucrose in PBS for 48–72 h, embedded in 1:1, 30% sucrose: OCT (Tissue-Tek OCT Compound 4583, Sakura Finetek), and sectioned coronally at 30 μm using a cryostat (Leica). For immunohistochemistry in thick sections, animals were perfused with PBS, brains were then dissected and sectioned coronally at 300–400 μm using a vibratome (Leica), and sections were then fixed with 4% PFA for 30 min. Cryosections or thick sections were then washed with PBS, blocked for 1 h at room temperature (10% normal donkey serum (NDS) and 0.3% Triton X-100 diluted in PBS), and incubated at 4 °C with primary antibodies in blocking solution. Cryosections were incubated overnight, whereas thick sections were incubated for 5 days. Primary antibodies used were: anti-NeuN (mouse, 1:500; ab104224, abcam) anti-CTIP2 (rat, 1:300; ab18465, abcam), anti-GFAP (rabbit, 1:1,000; Z0334, Dako), anti-GFP (chicken, 1:1,000; GTX13970, GeneTex), anti-HNA (mouse, 1:200; ab191181, abcam), anti-NeuN (rabbit, 1:500; ABN78, Millipore), anti-PDGFRA (rabbit, 1:200; sc-338, Santa Cruz), anti-PPP1R17 (rabbit, 1:200; HPA047819, Atlas Antibodies), anti-RECA-1 (mouse, 1:50; ab9774, abcam), anti-SCG2 (rabbit, 1:100; 20357-1-AP, Proteintech), anti-SOX9 (goat, 1:500; AF3075, R&D Systems), Netrin G1a (goat, 1:100; AF1166, R&D Systems), anti-STEM121 (mouse, 1:200; Y40410, Takara Bio), anti-SATB2 (mouse, 1:50; ab51502, abcam), anti-GAD65/67 (rabbit, 1:400; ABN904, Millipore) and anti-IBA1 (goat, 1:100; ab5076, abcam). Sections were then washed with PBS and incubated with secondary antibodies for either 1 h at room temperature (cryosections) or overnight at 4 °C (thick sections). Alexa Fluor secondary antibodies (Life Technologies) diluted in blocking solution at 1:1,000 were used. Following washes with PBS, nuclei were visualized with Hoechst 33258 (Life Technologies). Finally, slides were mounted for microscopy with cover glasses (Fisher Scientific) using Aquamount (Polysciences) and imaged on a Keyence fluorescence (BZ-X analyzer) microscope or Leica TCS SP8 (Las-X) confocal microscope. Images were processed in ImageJ (Fiji). To quantify the fraction of human neurons in t-hCO and the rat cortex, 387.5-µm-wide rectangular images were taken at the t-hCO centre, edge or from the adjacent rat cortex. The edge of the graft was determined by assessing changes in tissue transparency, the presence of HNA^+^ nuclei and/or tissue autofluorescence. In each image, the total number of NeuN^+^ and HNA^+^ cells was divided by the total number of NeuN^+^ cells within the same area. To ensure that only cells with nuclei in the imaging plane were counted, only cells that were also Hoechst^+^ were included in the calculation. Two images, taken at least 1 mm apart, were averaged to reduce statistical error.

### Single-nuclei dissociation and gene expression

One week before sample collection, animals transplanted with hCO (approximately 8 months of differentiation) were housed in a dark room and whiskers were trimmed to minimize sensory stimulation. Nuclei isolation was performed as previously described^31^ with some modifications. In brief, t-hCO and hCO were disrupted using the detergent-mechanical cell lysis method with a 2-ml glass tissue grinder (D8938, Sigma-Aldrich/KIMBLE). Crude nuclei were then filtered using a 40-μm filter and centrifuged at 320*g* for 10 min at 4 °C before performing a sucrose density gradient. After a centrifugation step (320*g* for 20 min at 4 °C), samples were resuspended in 0.04% BSA/PBS supplemented with 0.2 U μl^−1^ RNAse inhibitor (40 U μl^−1^, AM2682, Ambion) and passed through a 40-μm flowmi filter. Dissociated nuclei were then resuspended in PBS containing 0.02% BSA and loaded onto a Chromium Single cell 3′ chip (with an estimated recovery of 8,000 cells per channel). snRNA-seq libraries were prepared with the Chromium Single cell 3′ GEM, Library & Gel Bead Kit v3 (10x Genomics). Libraries from different samples were pooled and sequenced by Admera Health on a NovaSeq S4 (Illumina).

### Single-nuclei expression analysis

Gene expression levels were quantified for each putative nuclei barcode using the 10x Genomics CellRanger analysis software suite (version 6.1.2). Specifically, reads were mapped to a combined human (GRCh38, Ensemble release 98) and rat (Rnor_6.0, Ensemble release 100) reference genome created using the mkref command and quantified using the count command with –include-introns=TRUE to include reads mapping to intronic regions. For t-hCO samples, human nuclei were identified based on a conservative requirement of at least 95% of total mapped reads aligning to the human genome. All subsequent analyses were performed on the filtered barcode matrices outputted from CellRanger using the R (version 4.1.2) package Seurat (version 4.1.1)^32^.

To ensure that only high-quality nuclei were included for downstream analyses, an iterative filtering process was implemented for each sample. First, low-quality nuclei with less than 1,000 unique genes detected and with mitochondrial counts accounting for greater than 20% of the total counts were identified and removed. Subsequently, raw gene count matrices were normalized by regularized negative binomial regression using the sctransform function (vst.flavor=”v2”), which also identified the top 3,000 highly variable genes using default parameters. Dimensionality reduction using principal component analysis (PCA) on the top variable genes was performed, and clusters of nuclei were identified in PCA space by shared nearest-neighbour graph construction and modularity detection implemented by the FindNeighbors and FindClusters functions using a dataset dimension of 30 (dims = 30 chosen based on visual inspection of elbow plot and used for all samples and integration analyses) with default parameters. We subsequently performed iterative rounds of clustering (resolution = 1) to identify and remove clusters of putative low-quality cells based on outlier low gene counts (median below the 10th percentile), outlier high-fraction mitochondrial genes (median above the 95th percentile), and/or high proportions of putative doublets identified by the DoubletFinder package^33^ (median DoubletFinder score above the 95th percentile). t-hCO samples (*n* = 3) and hCO samples (*n* = 3) were each separately integrated using the IntegrateData function with the above parameters. Another round of quality filtering was subsequently performed on the integrated datasets as described above.

Following low-quality nuclei removal, integrated datasets were clustered (resolution = 0.5) and embedded for visualization purposes with UMAP^34^. Marker genes for each cluster were determined using the FindMarkers function with default parameters, calculated on the normalized gene expression data. We identified and categorized major cell classes through a combination of marker^19–21,35^ gene expression and annotation via integration with reference human fetal and adult cortical datasets. Specifically, cycling progenitors were identified by the expression of *MKI67* and *TOP2A*. The Progenitor clusters were defined by absence of mitotic transcripts, high cluster overlap to the multipotent glial progenitor cell cluster described in the late midfetal cortex^20^, and expression of *EGFR* and *OLIG1*. We used the term astroglia to encompass multiple states of astrocyte differentiation, from late radial glia through to astrocyte maturation. Astroglia clusters expressed high levels of *SLC1A3* and *AQP4* and demonstrated mapping to fetal radial glia subtypes and/or adult astrocytes. OPCs expressed *PDGFRA* and *SOX10*, whereas oligodendrocytes expressed markers of myelination (*MOG* and *MYRF*). Glutamatergic neurons were identified by the presence of neuronal transcripts (*SYT1* and *SNAP25*), absence of GABAergic markers (*GAD2*) and expression of *NEUROD6*, *SLC17A7*, *BCL11B* or *SATB2*. GluN neurons were further classified into upper layer (expression of *SATB2* and absence of *BCL11B*) and deep layer (expression of *BCL11B*) subclasses. Putative subplate (SP) neurons expressed known SP markers^18^ such as *ST18* and *SORCS1*, in addition to deep layer GluN markers. Choroid plexus-like cells were defined by expression of *TTR* and meningeal-like cells expressed fibroblast-associated genes and mapped to leptomeningeal/vascular cells of reference datasets.

Differential gene expression analysis between t-hCO and hCO subclasses was performed using a recently developed pseudobulk approach^36^ across sample replicates, implemented with the Libra R package (version 1.0.0). Specifically, the edgeR (version 3.36.0, R package) log-likelihood ratio test was performed between groups on gene counts summed across cells for a given cell class for each sample replicate. For heat map visualizations, counts per million (CPM) normalized expression values were calculated using edgeR (cpm() function) and scaled (to achieve mean = 0, standard deviation = 1). Gene Ontology (GO) enrichment analyses among significantly upregulated t-hCO GluN genes (Benjamini–Hochberg-adjusted *P* values less than 0.05, expressed in at least 10% of t-hCO GluN cells, and fold change increase of at least 2) were performed using the ToppGene Suite (https://toppgene.cchmc.org/)^37^. We used the ToppFun application with default parameters and reported Benjamini–Hochberg-adjusted *P* values calculated from hypergeometric tests from GO annotations.

To map our snRNA-seq clusters to annotated cell clusters from reference primary adult single-cell RNA-seq or snRNA-seq studies^19–22^, we performed a pairwise dataset integration approach. We used the SCTransform normalization (v2) workflow in Seurat to integrate and compare cluster overlap between datasets (using identical parameters described above). Individual datasets were randomly subsetted to have a maximum of 500 cells or nuclei per original cluster to improve computational efficiency. Using an analogous approach previously described^22^, cluster overlap was defined as the proportion of cells or nuclei in each integrated cluster that overlapped with the reference cluster labels. To further classify GluN, we utilized the TransferData workflow in Seurat on GluN subsetted data, to assign reference dataset labels to our GluN cells.

To assess the global transcriptomic maturation state of t-hCO and hCO samples, we compared our pseudobulk samples with BrainSpan/psychENCODE^23^, which consists of bulk RNA-seq spanning human brain development. We performed PCA on the combined sample normalized gene expression matrix from cortical samples of 10 postconception weeks and older, subsetted to 5,567 genes (shared with our data) that were previously identified to be developmentally regulated across BrainSpan cortical samples (defined as greater than 50% variance explained by age using a cubic model)^38^. We further obtained genes associated with major neurodevelopmental transcriptomic signatures through non-negative matrix factorization, as previously described^38^. Sample weights calculated from the non-negative matrix factorization procedure are plotted in Extended Data Fig. 5b for each of the five signatures described by Zhu et al.^38^. Similarly, activity-dependent transcriptional markers were obtained from previous published studies. Specifically, significantly upregulated ERGs and LRGs from glutamatergic neurons identified by snRNA-seq collection of the mouse visual cortex after visual stimulation were obtained from Supplementary Table 3 of Hrvatin et al.^16^. Human-enriched LRGs were obtained from human fetal brain cultures activated with KCl and collected 6 h after stimulation, which were filtered for genes significantly upregulated in humans but not rodents^17^ (Supplementary Table 4). Gene set enrichment analyses using these gene sets were performed using a one-sided Fisher’s exact test.

### Ex vivo t-hCO slice electrophysiology

Rats were anaesthetized with isoflurane and brains were removed and placed in cold (approximately 4 °C) oxygenated (95% O_2_ and 5% CO_2_) sucrose slicing solution containing: 234 mM sucrose, 11 mM glucose, 26 mM NaHCO_3_, 2.5 mM KCl, 1.25 mM NaH_2_PO_4_, 10 mM MgSO_4_ and 0.5 mM CaCl_2_ (approximately 310 mOsm). Coronal rat brain slices (300–400 µm), containing t-hCO, were sectioned using a Leica VT1200 vibratome as previously described^39^. Slices were then moved to a continuously oxygenated slice chamber at room temperature, which contained aCSF made with: 10 mM glucose, 26 mM NaHCO_3_, 2.5 mM KCl, 1.25 mM NaHPO_4_, 1 mM MgSO_4_, 2 mM CaCl_2_ and 126 mM NaCl (298 mOsm) for at least 45 min before recording. Slice recordings were performed in a submerged chamber where they were continuously perfused with aCSF (bubbled with 95% O_2_ and 5% CO_2_). All data were recorded at room temperature. t-hCO neurons were patched with a borosilicate glass pipette filled with an internal solution containing 127 mM potassium gluconate, 8 mM NaCl, 4 mM magnesium ATP, 0.3 mM sodium GTP, 10 mM HEPES and 0.6 mM EGTA, pH 7.2, adjusted with KOH (290 mOsm). For reconstruction purposes, biocytin (0.2%) was added to the recording solution.

Data were acquired with a MultiClamp 700B Amplifier (Molecular Devices) and a Digidata 1550B Digitizer (Molecular Devices), low-pass filtered at 2 kHz, digitized at 20 kHz and analysed using Clampfit (Molecular Devices), Origin (OriginPro 2021b, OriginLab) and custom MATLAB (Mathworks) functions. The liquid junction potential was calculated using JPCalc, and recordings were corrected with an estimated −14 mV. The *I*–*V* manipulations were constructed from a series of current steps in 10–25 pA increments from –250 to 750 pA.

Electrical stimulations of thalamic, white matter and S1 afferents during patch clamp recordings of hCO neurons were performed in thalamocortical slices, as previously described^25^. In brief, the brain was situated on a 3D-printed stage tilted at 10° and the frontal brain was cut with a 35° angle. The brain was then glued onto the cut surface, and slices, which preserve the thalamocortical projection axons, were generated. Bipolar tungsten electrodes (0.5 MΩ) were mounted on a second micromanipulator and were strategically positioned to stimulate four regions per each cell (internal capsule, white matter, S1 and hCO). Synaptic responses were recorded following a 300-µA phasic stimulation at 0.03–0.1 Hz.

hChR2-expressing hCO neurons were activated with 480 nm, LED-generated (Prizmatix) light pulses delivered via a ×40 objective (0.9 NA; Olympus) onto hChR2-expressing processes next to the recorded cell. The illumination field was approximately 0.5 mm in diameter at a total intensity of 10–20 mW. Pulse width was set at 10 ms, which corresponds to the pulse delivered during behavioural training experiments. Multiple stimulation frequencies were applied, from 1 to 20 Hz, but for quantification, only the first pulse of the train was used. The inter-train interval was typically more than 30 s long, to minimally affect synaptic depression or facilitation pathways. To test whether hChR2 responses are monosynaptic, we applied TTX (1 µM) to the bath until the EPSC response was eliminated, which was followed by application of 4-amino-pyridine (4-AP; 100 µM). Typically, the response was recovered within several minutes, with a slightly longer delay between LED onset and EPSC generation. NBQX (10 µM) was applied to test whether the responses were driven by AMPA receptors.

### Slice preparation and patch clamp recordings of in vitro hCO

Acute hCO slices were generated as previously described^5^. In brief, hCO slices were embedded in 4% agarose and transferred to an aCSF containing 126 mM NaCl, 2.5 mM KCl, 1.25 mM NaH_2_PO_4_, 1 mM MgSO_4_, 2 mM CaCl_2_, 26 mM NaHCO_3_ and 10 mM d-(+)-glucose. Slices were cut at 200–300 µm at room temperature using a Leica VT1200 vibratome and maintained in aCSF at room temperature. Whole-cell patch clamp recordings from hCO slices were then performed under an upright SliceScope microscope (Scientifica). Slices were perfused with aCSF (bubbled with 95% O_2_ and 5% CO_2_), and signals from cells were recorded at room temperature. hCO neurons were patched with a borosilicate glass pipette filled with an internal solution containing 127 mM potassium gluconate, 8 mM NaCl, 4 mM magnesium ATP, 0.3 mM sodium GTP, 10 mM HEPES and 0.6 mM EGTA, pH 7.2, adjusted with KOH (290 mOsm). For reconstruction purposes, 0.2% biocytin was added to the internal solution.

Data were acquired with Clampex (Clampex 11.1, Molecular Devices) using a MultiClamp 700B Amplifier (Molecular Devices) and a Digidata 1550B Digitizer (Molecular Devices), low-pass filtered at 2 kHz, digitized at 20 kHz and analysed using Clampfit (version 10.6, Molecular Devices) and custom MATLAB (MATLAB 2019b, Mathworks) functions. The liquid junction potential was calculated using JPCalc, and recordings were corrected with an estimated −14 mV liquid junction potential. The *I*–*V* manipulations were constructed from a series of current steps in 5–10-pA increments from –50 to 250 pA.

### Streptavidin staining and neuron tracing

For morphological reconstruction of patch-clamped neurons, 0.2% biocytin (Sigma-Aldrich) was added to the internal solution. Cells were filled for at least 15 min after break-in. Pipettes were then retracted slowly, over 1–2 min, until the recorded membrane fully resealed. Following slice physiology procedures, slices were post-fixed in 4% PFA overnight at 4 °C and then washed with PBS X3 before being incubated with streptavidin-conjugated DyLight 549 or DyLight 405 (Vector Labs) at 1:1,000 dilution for 2 h at room temperature to label cells that were filled with biocytin (2%; Sigma-Aldrich) during patch clamp recordings. Slices were then mounted for microscopy on glass slides using Aquamount (Thermo Scientific) and imaged the next day on Leica TCS SP8 confocal microscope, using a ×40 1.3 NA, oil immersion objective, at ×0.9–1.0 zoom with an *xy* sampling frequency of approximately 7 pixels per micrometre. Z stacks at 1-μm intervals were serially obtained and z-stack tiling and Leica-based automated stitching were performed to cover the entire dendritic tree of each neuron. The neurons were subsequently semi-manually traced using the neuTube interface^40^ and SWC files were generated. The files were next loaded into Fiji (ImageJ, version 2.1.0; NIH) plugin SimpleNeuriteTracer^41^.

### Primary human sample collection

Human cerebral cortical tissue was obtained with informed consent under a protocol approved by the Stanford University Institutional Review Board. The two postnatal human tissue samples (3 and 18 years of age) were obtained from resection of the frontal lobe cortex (middle frontal gyrus) as part of surgeries for treating medically refractory epilepsy. After resection, tissue was collected in ice-cold NMDG-aCSF containing: 92 mM NMDG, 2.5 mM KCl, 1.25 mM NaH_2_PO_4_, 30 mM NaHCO_3_, 20 mM HEPES, 25 mM glucose, 2 mM thiourea, 5 mM Na-ascorbate, 3 mM Na-pyruvate, 0.5 mM CaCl_2_·4H_2_O and 10 mM MgSO_4_·7H_2_O. The pH was titred to 7.3–7.4 with concentrated hydrochloric acid. The tissue was transferred to the laboratory within 30 min and coronal sections were made per the procedure described above.

### Fibre implantation for fibre photometry

All animal procedures followed animal care guidelines approved by Stanford University’s APLAC. Rats (more than 140 days after transplantation) were anaesthetized with 5% isoflurane for induction and 1–3% isoflurane during surgery. Animals were placed into a stereotactic frame (Kopf) and Buprenorphine Sustained-Release (SR) was administered subcutaneously. The skull was exposed, cleaned and 3–5 bone screws were inserted. To target the t-hCO, we generated stereotactic coordinates based on the images acquired with MRI. A burr hole was drilled at the site of interest, and a fibre (400 μm in diameter, 0.48 NA, Doric) was lowered to 100 µm below the surface of the hCO and affixed to the skull with UV-cured dental cement (Relyx).

### Fibre photometry recordings

Fibre photometry recordings were performed as previously described^42^. For recordings of spontaneous activity, rats were placed into a clean homecage and a 400-µm diameter fibre-optic patch cord (Doric) coupled to the fibre photometry acquisition system was connected to the implanted optical fibre. Animals were free to explore the homecage during a 10-min recording of spontaneous activity. For recordings of evoked activity, rats (more than 140 days after transplantation) were anaesthetized with 5% isoflurane for induction and 1–3% isoflurane for maintenance. Animals were placed into a stereotactic frame (Kopf), and whiskers contralateral to the t-hCO were trimmed to approximately 2 cm and threaded through mesh that was coupled to a piezo-electric actuator (PI). A 400-µm diameter fibre-optic patch cord (Doric) coupled to the acquisition system was connected to the implanted optical fibre. Fifty deflections (2 mm at 20 Hz for 2 s per presentation) were then made to the whiskers contralateral to the t-hCO using a piezo-electric actuator at random times during a 20-min recording. Deflection timing was controlled using custom MATLAB code using the MATLAB Support Package for Arduino. Events were synchronized with the acquisition software using transistor-transistor logic (TTL) pulses.

### Cranial window surgery

Rats (more than 140 days after transplantation) were anaesthetized with 5% isoflurane for induction and 1–3% isoflurane during surgery. Animals were placed into a stereotactic frame (Kopf), and Buprenorphine SR and dexamethasone were administered subcutaneously. The skull was exposed, cleaned and 3–5 bone screws were inserted. To target t-hCO, we generated stereotactic coordinates based on the images acquired with MRI. A circular craniotomy (approximately 1 cm in diameter) was made with a high-speed drill directly above the transplanted hCO. Once the bone was as thin as possible, but before drilling all the way through the bone, the remaining intact bone disk was removed using forceps to reveal the underlying t-hCO. The craniotomy was filled with sterile saline, and a glass coverslip and custom headbar were affixed to the skull with UV-cured dental cement (Relyx).

### Acute in vivo two-photon calcium imaging

Two-photon imaging was performed using a Bruker multiphoton microscope using Nikon LWD objective (×16, 0.8 NA). Imaging of GCaMP6 was conducted at 920 nm, ×1.4 zoom in a single *z*-plane, with 8× frame averaging at 7.5 frames per second. Rats were anaesthetized with 5% isoflurane for induction and maintained with 1–3% isoflurane. Rats were placed into a custom head-fixed apparatus and positioned beneath the objective. A 3-min baseline recording of spontaneous activity was obtained. Fifty airpuffs (100-ms duration per presentation) were then delivered to the whisker pad contralateral to the t-hCO using a picospritzer at random times during a 20-min recording. Airpuff timing was controlled using custom MATLAB code using the MATLAB Support Package for Arduino. Events were synchronized with the acquisition software (PrairieView 5.5) using TTL pulses. For analysis, images were corrected for *x*–*y* motion using affine corrections in MoCo, running in Fiji. Fluorescence traces from individual cells were extracted using CNMF-E^43^. Fluorescence from each region of interest was extracted, converted to a dF/F trace, and then converted into a z-score.

### Acute in vivo extracellular electrophysiology

Rats (more than 140 days after transplantation) were anaesthetized with 5% isoflurane for induction and 1–3% isoflurane during surgery. Animals were placed into a stereotactic frame (Kopf) and Buprenorphine SR and dexamethasone were administered subcutaneously. Whiskers contralateral to the t-hCO were trimmed to approximately 2 cm and threaded through mesh that was coupled to a piezo-electric actuator. The skull was exposed and cleaned. A stainless-steel ground screw was fastened to the skull. To target the t-hCO, we generated stereotactic coordinates based on the images acquired with MRI. A circular craniotomy (approximately 1 cm in diameter) was made with a high-speed drill directly above the t-hCO. Once the bone was as thin as possible, but before drilling all the way through the bone, the remaining intact bone disk was removed using forceps to reveal the underlying t-hCO. Single units were recorded using either 32-channel or 64-channel high-density silicon probes (Cambridge Neurotech) grounded to the ground screw and pre-amplified with an RHD amplifier (Intan). Electrodes were lowered through the craniotomy into the target site using a manipulator and the craniotomy was filled with sterile saline. Data acquisition was performed at 30 kHz with an Open Ephys acquisition system. Recordings only proceeded if we identified more than ten channels with highly correlated, rhythmic spontaneous activity, suggesting that electrodes were positioned in the graft (based on two-photon calcium imaging data). A 10-min baseline recording of spontaneous activity was obtained. Fifty deflections (2 mm at 20 Hz for 2 s per presentation) were then made to the whiskers contralateral to the t-hCO using a piezo-electric actuator at random times during a 20-min recording. Deflection timing was controlled using custom MATLAB code using the MATLAB Support Package for Arduino (MATLAB 2019b). Events were synchronized with the acquisition software using TTL pulses.

For optotagging experiments, a 200-µm diameter fibre-optic patch cord (Doric), coupled to a 473-nm laser (Omicron), was connected to a 200-µm diameter optical fibre that was positioned above the craniotomy. Immediately before this, the power output from the patch cord was adjusted to 20 mW. Electrodes were lowered through the craniotomy into the target site using a manipulator and the craniotomy was filled with sterile saline. Ten pulses of 473-nm light (at 2 Hz with a 10-ms pulse width) were delivered at the start of the recording. Light-responsive units were defined as units that displayed a spiking response within 10 ms of light onset on 70% or more of trials.

For analysis, spikes were sorted using Kilosort2 and were manually curated using Phy2 (ref. ^44^). Firing rates were computed using 200-ms bins, with a sliding window of 100 ms and converted into a z-score. A hidden Markov model with two states was used to label ‘on’ and ‘off’ states in the population activity. On states were considered to represent bursts, and off states were considered to represent inter-burst intervals. The emission transition parameters of the model were fit using the Baum–Welch algorithm (MATLAB hmmtrain with a convergence threshold of 1 × 10^–6^ and initial guesses of transition matrix: [0.95, 0.05; 0.05, 0.96] and emission: [0.5, 0.5; 0.1, 0.99]), and the state assignment at each time point was then estimated using the Viterbi algorithm. To assess responses to whisker deflection, a Wilcoxon signed-rank test was performed to compare firing rates in the 1 s following the onset of whisker deflection to the 1 s before whisker deflection with a significance threshold of *P* < 0.05. Latencies were computed as the time to reach peak z-score in the 2 s following whisker deflection. The power spectral density was calculated using Welch’s method (pwelch() in MATLAB), with a window slide of 10 × fs, where fs is the sampling rate of the signal.

### Fibre implantation for optogenetic manipulations

Rats (more than 90 days after transplantation) were anaesthetized with 5% isoflurane for induction and 1–3% isoflurane during surgery. Animals were placed into a stereotactic frame (Kopf) and Buprenorphine SR was administered subcutaneously. The skull was exposed, cleaned and 3–5 bone screws were inserted. To target the t-hCO, we generated stereotactic coordinates based on the images acquired with MRI. A burr hole was drilled at the site of interest, and a fibre (200 μm in diameter, 0.48 NA; Thorlabs) was lowered to 100 µm below the surface of the hCO and affixed to the skull with UV-cured dental cement (Relyx).

### Optogenetic behavioural assay

Water scheduled rats (1 h of water per day) were placed into a custom operant chamber containing a nosepoke portal equipped with a lick spout for water reward delivery. Entries into the nosepoke portal were detected by the breakage of an infrared beam, and licks were detected using a capacitive touch sensor. All events were controlled and recorded using custom MATLAB code using the MATLAB Support Package for Arduino (MATLAB 2019b). At least 1 week after surgery, animals began pre-training. On day 1 of pre-training, animals received small water rewards at the reward spout at randomized delays for 1 h. On days 2 and 3 of pre-training, animals received small water rewards only after performing increasing numbers of licks to the lick spouts for 1 h. All animals readily performed this behaviour. After pre-training, animals were trained to associate optogenetic stimulation of the transplanted hCO with reward delivery. Animals were placed into the operant chamber and a 200-µm diameter fibre-optic patch cord (Doric), coupled to both a 473-nm (Omicron) and a 635-nm (CNI) laser outside of the operant chamber, was connected to the implanted optical fibre. Immediately before this, the power output from the patch cord was adjusted to 20 mW. Laser timing was controlled by a Master-8 pulse generator (AMPI). One second after entering the nosepoke portal, animals received random presentations of either 473-nm or 635-nm stimulation (10 Hz with 10-ms pulse width for 5-s total stimulation). If animals performed one or more licks during the 473-nm stimulation, a small water reward was dispensed at the reward spout after stimulation was complete. The next trial was initiated after collection of this reward. If animals performed one or more licks during the 635-nm stimulation, there was no consequence. Trials were separated by a variable interval of 5–10 s. Animals received daily training sessions that concluded after 150 473-nm and 150 635-nm trials had been completed or after 150 min, whichever came first, for a total of 15 days. Behavioural performance was quantified by calculating a preference index for each training session: (number of licks during 473-nm stimulation − number of licks during 635-nm stimulation)/(number of licks during 473-nm stimulation + number of licks during 635-nm stimulation). The learning success rate was defined as the percentage of trained animals that achieved a preference score >0.2 on at least 2 consecutive training days.

### Optogenetic stimulation and FOS staining

Rats were placed into a clean rat cage, and a 200-µm diameter fibre-optic patch cord (Doric), coupled to a 473-nm (Omicron) laser, was connected to the implanted optical fibre. Immediately before this, the power output from the patch cord was adjusted to 20 mW. Stimulated animals received 10 Hz, 10-ms pulse width and 473-nm stimulation for 10 min before being returned to their homecage. Unstimulated animals received no stimulation for 10 min before being returned to their homecage. Rats were euthanized by transcardial perfusion with 150 ml PBS, followed by 100 ml 4% PFA 90 min after optogenetic stimulation. Brains were extracted and 100-μm sections were cut on a vibratome. The slices were labelled with goat anti-GFP (abcam) and rabbit anti-FOS (abcam) primary antibodies, Alexa 488 donkey anti-goat (Invitrogen) and Alexa 594 donkey anti-rabbit (Invitrogen) secondary antibodies, and DAPI. Images were acquired using a confocal microscope (Zeiss) with a ×20 objective and overlaid with images from the Paxinos, George and Watson rat brain atlas for blinded manual counting of FOS-positive cells in specified brain regions.

### EEG recordings

*FOXN1*^−/−^ rats were anaesthetized with isoflurane and stereotactically implanted with stainless steel wires (791400, A-M Systems) over the bilateral somatosensory cortices and the bilateral motor cortices. A reference wire was positioned over the cerebellum, and implants were secured with dental cement (Metabond, S399, S371 and S398; Jet Set4 Liquid, Lang Dental, 3802X6). The following stereotactic coordinates were used, relative to Bregma: primary somatosensory cortex (S1BF), anterior–posterior −1.3 mm and lateral 3.3 mm; and primary motor cortex (M1), anterior–posterior +2.5 mm, lateral 2.5 mm. The wires of the implant were secured onto custom-made Mill-Max headpiece adapters (ED90267-ND, Digi-Key Electronics). To initiate the recording, the adapters were connected to the head stage, consisting of a digitizer and amplifier board (C3334, Intan Technologies). Awake, freely behaving animals were tethered to an acquisition board (Open Ephys) with lightweight SPI interface cables (C3206, Intan Technologies). Continuous real-time EEG was recorded with Open Ephys software (version 0.4.4.1; https://open-ephys.org). Data were sampled at 2 kHz and bandpass filtered between 1 and 300 Hz.

### Assessment of locomotor behaviour in the open field arena

Rats (more than 3 months old, more than 90 days post-transplantation) were handled for 3 min on 5 consecutive days before behavioural testing began. Rats were placed into the corner of an open field activity arena (43 cm × 43 cm × 30 cm) containing three planes of infrared detectors within a sound attenuating chamber (Med Associates). Rats were allowed to explore the arena for 10 min, and the distance moved was computed with Med Associates software. The arena was cleaned with a 1% Virkon solution at the end of each session.

### Novel object recognition

Two different objects (green tower and white bottle) were used in this test. The objects had similar heights and volumes, but differed in their shape, texture and appearance. On day 1, rats were placed into a black square plastic area (50 cm × 50 cm × 45 cm) and allowed to explore the arena with two habituation objects (15-ml centrifuge tubes) for 5 min. On day 2, rats first underwent a training session. In this session, rats were placed into the arena and allowed to explore the arena for 5 min with two identical objects, which were placed in diagonally opposite corners 15 cm away from the corner. Rats were then returned to their homecage for 5 min. For the testing session, rats were placed back into the arena for 5 min and one of the two objects was replaced with a novel object. Rats were tracked with an automated tracking system (Noldus Information Technology), and time spent interacting with each object was manually scored by an experimenter who was blind to the experimental groups. Interaction was defined as the rat pointing its nose towards the object within 2 cm of it. Objects for training and testing and the location of these objects were pseudorandomized. Objects and the arena were cleaned with a 1% Virkon solution at the end of each training and testing session. A discrimination index was calculated as (time spent interacting with novel object – time spent interacting with familiar object)/(time spent interacting with novel object + time spent interacting with familiar object) during the testing session.

### Fear conditioning

The experiment consisted of 1 day of training, 1 day of contextual fear testing and 1 day of cued fear testing. The same context was used for both training and contextual testing. This context had aluminium walls, a grey metal grid for a floor, yellow house lights and the scent of mint extract, and was cleaned with 10% simple green solution between rats (Coulbourn Instruments). The cued fear testing context was circular with plastic walls and floor, blue house lights and the scent of vanilla extract, and was cleaned with 70% ethanol between rats. On day 1 (training), rats were placed individually into the training chamber for 200 s. A tone (20 s, 80 dB, 2 kHz) was presented followed by an electrical shock (0.5 mA for a duration of 2 s) 18 s after the end of the tone. This procedure was repeated a total of three times with 60-s intervals between the end of the shock and the start of the subsequent tone. Rats were removed from the chamber and returned to their homecage 60 s after the last shock. On day 2, rats were placed back in the training context without any tone or shock for 5 min for contextual memory testing. On day 3, the rats were placed in the cued fear context. After 200 s of habituation, rats were presented with tones (20 s, 80 dB, 2 kHz) three times at 80-s intervals. Stimulus presentation was controlled using FreezeFrame software. An overhead camera was used to record behaviour. Freezing behaviour was scored manually by an experimenter who was blind to the experimental groups. Freezing was defined as the absence of all movements except those caused by respiration.

### Statistics and reproducibility

Data are presented as mean ± s.e.m., unless otherwise indicated. Distribution of the raw data was tested for normality of distribution; statistical analyses were performed using the Student’s *t*-test, Kolmogorov–Smirnov test, Wilcoxon signed-rank test or analysis of variance with Bonferroni correction for multiple comparisons. Statistical analyses were performed in Prism (GraphPad) and OriginPro (OriginLab). Full statistical details for each figure panel are included in Supplementary Table 7. Data shown from representative experiments were repeated with similar results in at least three independent biological replicates, unless otherwise noted. Sample sizes were estimated empirically based on previous studies. hCO were randomly assigned to different experiments. Behavioural experiments were performed by researchers who were blinded to the experimental conditions.

### Ethics statement

All experiments involving human cells complied with all relevant guidelines and regulations. Human donors in this study consented to the use of their cells to generate hiPS cells and derived cells. The source of the cells and their institutional approvals are listed in Supplementary Table 1. This study also benefitted from a consultation with the Stanford Center for Law and the Biosciences on the ethical aspects of the work as part of the Stanford Big Idea Project on Brain Organogenesis (Stanford Wu Tsai Neurosciences Institute). Approval for transplantation of hCO into rats was obtained from the Stanford Laboratory Animal Care (APLAC) Research Compliance Office. No discernible locomotor or memory deficits were detected in transplanted animals and their wellbeing was monitored throughout. Surgical neural tissue samples were obtained with approval from the Stanford University Institutional Review Board.

### Reporting summary

Further information on research design is available in the Nature Research Reporting Summary linked to this article.

## Online content

Any methods, additional references, Nature Research reporting summaries, source data, extended data, supplementary information, acknowledgements, peer review information; details of author contributions and competing interests; and statements of data and code availability are available at 10.1038/s41586-022-05277-w.

## Supplementary information

Reporting Summary

Supplementary Table 1 hiPS cell lines used for experiments. hiPS cell lines used in various experiments

Supplementary Table 2 Morbidity reports submitted by veterinary staff for transplanted and non-transplanted animals over a 12-month period.

Supplementary Table 3 Transplanted hCO cell type cluster marker genes. Marker genes for each annotated cell cluster form the integrated t-hCO snRNA-seq determined using the Seurat FindMarkers function with default parameters (two-sided Wilcoxon Rank Sum test with Bonferroni correction).

Supplementary Table 4 hCO cell type cluster marker genes. Marker genes for each annotated cell cluster form the hCO snRNA-seq dataset determined using the Seurat FindMarkers function with default parameters (two-sided Wilcoxon Rank Sum test with Bonferroni correction).

Supplementary Table 5 Glutamatergic neuron differentially expressed genes. Significantly increased differentially expressed t-hCO GluN genes (EdgeR log likelihood ratio test with Benjamini-Hochberg adjustment, adjusted *P* value < 0.05, expressed in at least 10% of t-hCO GluN cells) with absolute fold change difference of at least 2 between t-hCO and hCO glutamatergic neurons.

Supplementary Table 6 Gene ontology (GO) term enrichment for up-regulated genes in t-hCO glutamatergic neurons. GO term enrichment *P* values (hypergeometric test) of up-regulated genes in t-hCO glutamatergic neurons compared to hCO glutamatergic neurons (Supplementary Table 5)

Supplementary Table 7 Statistical details for Figures 1–5, Extended Data Figures 1–10.

Supplementary Video 1 In vivo two-photon calcium imaging of spontaneous activity of t-hCO.

Supplementary Video 2 Optogenetic activation of t-hCO drives reward-seeking behaviour.

## Figures and Tables

**Extended Data Fig. 2:**
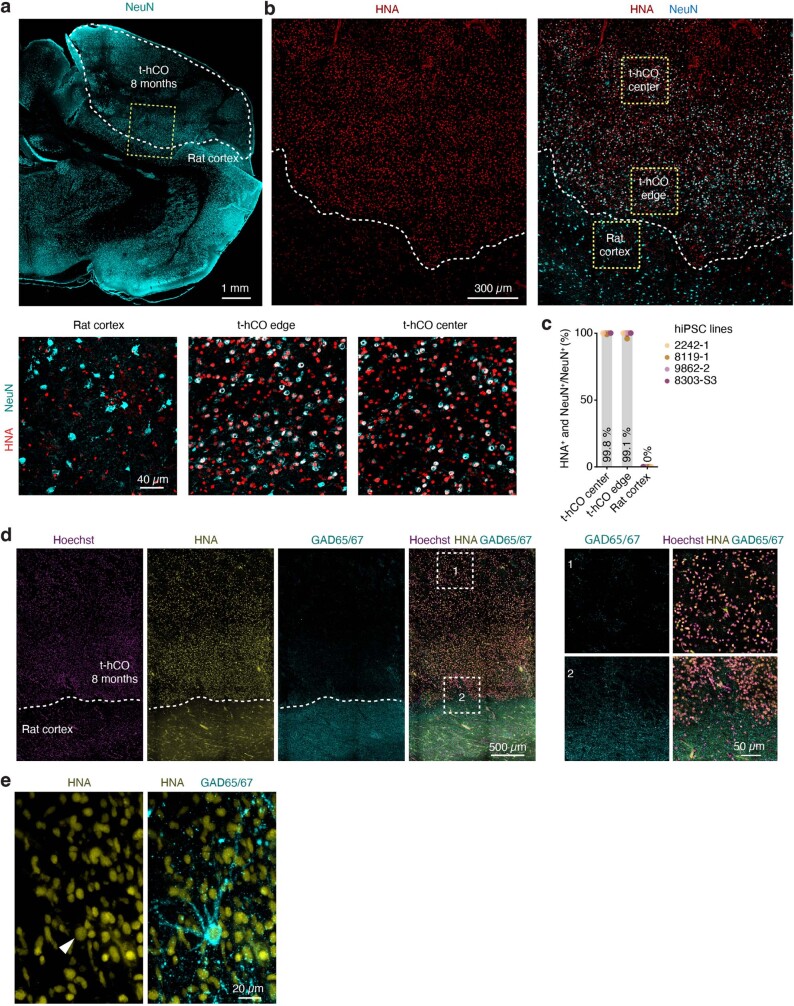
Immunohistochemical characterization of t-hCO. **a–b**. NeuN expression in t-hCO and the surrounding rat brain. **c**. Quantification of the overlap of HNA and NeuN expression in t-hCO and rat cortex (n = 5 t-hCO from 4 hiPS lines, 1–2 t-hCO per line). **d**. Representative images of GAD65/67 expression in t-hCO-rat cortex border. **e**. Example image of a rarely observed HNA^+^GAD65/67^+^ neuron in t-hCO. All images were acquired 8 months after transplantation unless otherwise stated.

## Data Availability

Single-cell gene expression data are available under the Gene Expression Omnibus accession number GSE190815. The data that support the findings of this study are available on request from the corresponding author. The following public datasets were used for snRNA-seq analysis: human genome sequence information from Ensembl (http://ftp.ensembl.org/pub/release-98/fasta/homo_sapiens/dna/Homo_sapiens.GRCh38.dna.primary_assembly.fa.gz) and human gene annotation from GENCODE (http://ftp.ebi.ac.uk/pub/databases/gencode/Gencode_human/release_32/gencode.v32.primary_assembly.annotation.gtf.gz); information on the rat genome sequence is from Ensembl (ftp://ftp.ensembl.org/pub/release-100/fasta/rattus_norvegicus/dna/Rattus_norvegicus.Rnor_6.0.dna.toplevel.fa.gz); the Allen Brain Institute generated human adult snRNA-seq data from the medial temporal gyrus^21^ and the primary motor cortex^22^ (https://portal.brain-map.org/atlases-and-data/rnaseq; accessed May 2022); human fetal cortical single-cell RNA-seq data^19,20^ (Polioudakis et al.^19^ data were obtained from http://solo.bmap.ucla.edu/shiny/webapp/ on April 2022; Trevino et al.^20^ data were downloaded from the Gene Expression Omnibus with the accession number GSE162170) and bulk RNA-seq data from the developing human cortex were generated by psychENCODE^38^ (http://development.psychencode.org/; accessed April 2022).

## Code availability

Data and code used for data processing and analysis are available on request from the corresponding author. The code used to analyse snRNA-seq data is available for download from https://github.com/kkelley85/Transplant_organoid_snRNAseq.

## References

[1] Kelley, K. W. & Pașca, S. P. Human brain organogenesis: toward a cellular understanding of development and disease. *Cell* **185**, 42–61 (2021). 34774127 10.1016/j.cell.2021.10.003 PMC13523617

[2] Pasca, A. M. et al. Functional cortical neurons and astrocytes from human pluripotent stem cells in 3D culture. *Nat. Methods* **12**, 671–678 (2015). 26005811 10.1038/nmeth.3415 PMC4489980

[3] Valesco, S. et al. Individual brain organoids reproducibly form cell diversity of the human cerebral cortex. *Nature* **570**, 523–527 (2019). 31168097 10.1038/s41586-019-1289-x PMC6906116

[4] Qian, X. et al. Brain-region-specific organoids using mini-bioreactors for modeling ZIKV exposure. *Cell* **165**, 1238–1254 (2016). 27118425 10.1016/j.cell.2016.04.032 PMC4900885

[5] Yoon, S. J. et al. Reliability of human cortical organoid generation. *Nat. Methods* **16**, 75–78 (2019). 30573846 10.1038/s41592-018-0255-0 PMC6677388

[6] Birey, F. et al. Assembly of functionally integrated human forebrain spheroids. *Nature* **545**, 54–59 (2017). 28445465 10.1038/nature22330 PMC5805137

[7] Espuny-Camacho, I. et al. Pyramidal neurons derived from human pluripotent stem cells integrate efficiently into mouse brain circuits in vivo. *Neuron* **77**, 440–456 (2013). 23395372 10.1016/j.neuron.2012.12.011

[8] Linaro, D. et al. Xenotransplanted human cortical neurons reveal species-specific development and functional integration into mouse visual circuits. *Neuron* **104**, 972–986.e6 (2019). 31761708 10.1016/j.neuron.2019.10.002 PMC6899440

[9] Mansour, A. A. et al. An in vivo model of functional and vascularized human brain organoids. *Nat. Biotechnol.* **36**, 432–441 (2018). 29658944 10.1038/nbt.4127 PMC6331203

[10] Real, R. et al. In vivo modeling of human neuron dynamics and down syndrome. *Science* **362**, eaau1810 (2018). 30309905 10.1126/science.aau1810 PMC6570619

[11] Kitahara, T. et al. Axonal extensions along corticospinal tracts from transplanted human cerebral organoids. *Stem Cell Rep.* **15**, 467–481 (2020). 10.1016/j.stemcr.2020.06.016 PMC7419717 32679062

[12] Xiong, M. et al. Human stem cell-derived neurons repair circuits and restore neural function. *Cell Stem Cell* **28**, 112–126.e6 (2021). 32966778 10.1016/j.stem.2020.08.014 PMC7796915

[13] Kichula, E. A. & Huntley, G. W. Developmental and comparative aspects of posterior medial thalamocortical innervation of the barrel cortex in mice and rats. *J. Comp. Neurol.* **509**, 239–258 (2008). 18496871 10.1002/cne.21690 PMC4913357

[14] Ebert, D. H. & Greenberg, M. E. Activity-dependent neuronal signalling and autism spectrum disorder. *Nature* **493**, 327–337 (2013). 23325215 10.1038/nature11860 PMC3576027

[15] Trujillo, C. A. et al. Complex oscillatory waves emerging from cortical organoids model early human brain network development. *Cell Stem Cell* **25**, 558–569.e7 (2019). 31474560 10.1016/j.stem.2019.08.002 PMC6778040

[16] Hrvatin, S. et al. Single-cell analysis of experience-dependent transcriptomic states in the mouse visual cortex. *Nat. Neurosci.* **21**, 120–129 (2018). 29230054 10.1038/s41593-017-0029-5 PMC5742025

[17] Ataman, B. et al. Evolution of osteocrin as an activity-regulated factor in the primate brain. *Nature* **539**, 120–129 (2016). 10.1038/nature20111 PMC5499253 27830782

[18] Hong, E. J., McCord, A. E. & Greenberg, M. E. A biological function for the neuronal activity-dependent component of Bdnf transcription in the development of cortical inhibition. *Neuron* **60**, 610–624 (2008). 19038219 10.1016/j.neuron.2008.09.024 PMC2873221

[19] Polioudakis, D. et al. A single-cell transcriptomic atlas of human neocortical development during mid-gestation. *Neuron* **103**, 785–801.e8 (2019). 31303374 10.1016/j.neuron.2019.06.011 PMC6831089

[20] Trevino, A. E. et al. Chromatin and gene-regulatory dynamics of the developing human cerebral cortex at single-cell resolution. *Cell* **184**, 5053–5069.e23 (2021). 34390642 10.1016/j.cell.2021.07.039

[21] Hodge, R. D. et al. Conserved cell types with divergent features in human versus mouse cortex. *Nature* **573**, 61–68 (2019). 31435019 10.1038/s41586-019-1506-7 PMC6919571

[22] Bakken, T. E. et al. Comparative cellular analysis of motor cortex in human, marmoset and mouse. *Nature* **598**, 111–119 (2021). 34616062 10.1038/s41586-021-03465-8 PMC8494640

[23] Li, M. et al. Integrative functional genomic analysis of human brain development and neuropsychiatric risks. *Science* **362**, eaat7615 (2018). 30545854 10.1126/science.aat7615 PMC6413317

[24] Gordon, A. et al. Long-term maturation of human cortical organoids matches key early postnatal transitions. *Nat. Neurosci.* **24**, 331–342 (2021). 33619405 10.1038/s41593-021-00802-y PMC8109149

[25] Krey, J. F. et al. Timothy syndrome is associated with activity-dependent dendritic retraction in rodent and human neurons. *Nat. Neurosci.* **16**, 201–209 (2013). 23313911 10.1038/nn.3307 PMC3568452

[26] Agmon, A. & Connors, B. W. Thalamocortical responses of mouse somatosensory (barrel) cortex in vitro. *Neuroscience* **41**, 365–379 (1991). 1870696 10.1016/0306-4522(91)90333-j

[27] Petreanu, L., Mao, T., Sternson, S. M. & Svoboda, K. The subcellular organization of neocortical excitatory connections. *Nature* **457**, 1142–1145 (2009). 19151697 10.1038/nature07709 PMC2745650

[28] Kalmbach, B. E. et al. h-Channels contribute to divergent intrinsic membrane properties of supragranular pyramidal neurons in human versus mouse cerebral cortex. *Neuron* **100**, 1194–1208.e5 (2018). 30392798 10.1016/j.neuron.2018.10.012 PMC6447369

[29] Molnár, Z., Luhmann, H. J. & Kanold, P. O. Transient cortical circuits match spontaneous and sensory-driven activity during development. *Science* **370**, eabb2153 (2020). 33060328 10.1126/science.abb2153 PMC8050953

[30] Jabaudon, D. Fate and freedom in developing neocortical circuits. *Nat. Commun.* **8**, 16042 (2017). 28671189 10.1038/ncomms16042 PMC5500875

[31] Matson, K. J. et al. Isolation of adult spinal cord nuclei for massively parallel single-nucleus RNA sequencing. *J. Vis. Exp.* **140**, 58413 (2018). 10.3791/58413 PMC6235529 30371670

[32] Stuart, T. et al. Comprehensive integration of single-cell data. *Cell* **177**, 1888–1902.e21 (2019). 31178118 10.1016/j.cell.2019.05.031 PMC6687398

[33] McGinnis, C. S., Murrow, L. M. & Gartner, Z. J. DoubletFinder: doublet detection in single-cell RNA sequencing data using artificial nearest neighbors. *Cell Syst.* **8**, 329–337.e4 (2019). 30954475 10.1016/j.cels.2019.03.003 PMC6853612

[34] Becht, E. et al. Dimensionality reduction for visualizing single-cell data using UMAP. *Nat. Biotechnol.* **37**, 38–44 (2019). 10.1038/nbt.4314 30531897

[35] Nowakowski, T. J. et al. Spatiotemporal gene expression trajectories reveal developmental hierarchies of the human cortex. *Science* **358**, 1318–1323 (2017). 29217575 10.1126/science.aap8809 PMC5991609

[36] Squair, J. W. et al. Confronting false discoveries in single-cell differential expression. *Nat. Commun.* **12**, 5692 (2021). 34584091 10.1038/s41467-021-25960-2 PMC8479118

[37] Chen, J., Bardes, E. E., Aronow, B. J. & Jegga, A. G. ToppGene Suite for gene list enrichment analysis and candidate gene prioritization. *Nucleic Acids Res.* **37**, W305–W311 (2009). 19465376 10.1093/nar/gkp427 PMC2703978

[38] Zhu, Y. et al. Spatiotemporal transcriptomic divergence across human and macaque brain development. *Science* **362**, eaat8077 (2018). 30545855 10.1126/science.aat8077 PMC6900982

[39] Huguenard, J. R. & Prince, D. A. Intrathalamic rhythmicity studied in vitro: nominal T-current modulation causes robust antioscillatory effects. *J. Neurosci.* **14**, 5485–5502 (1994). 8083749 10.1523/JNEUROSCI.14-09-05485.1994 PMC6577071

[40] Feng, L., Zhao, T. & Kim, J. Neutube 1.0: a new design for efficient neuron reconstruction software based on the SWC format. *eNeuro* **2**, ENEURO.0049-14.2014 (2015). 26464967 10.1523/ENEURO.0049-14.2014 PMC4586918

[41] Arshadi, C., Günther, U., Eddison, M., Harrington, K. I. S. & Ferreira, T. A. SNT: a unifying toolbox for quantification of neuronal anatomy. *Nat. Methods* **18**, 374–377 (2021). 33795878 10.1038/s41592-021-01105-7

[42] Steinberg, E. E. et al. Amygdala–midbrain connections modulate appetitive and aversive learning. *Neuron* **106**, 1026–1043.e9 (2020). 32294466 10.1016/j.neuron.2020.03.016

[43] Zhou, P. et al. Efficient and accurate extraction of in vivo calcium signals from microendoscopic video data. *eLife* **7**, e28728 (2018). 29469809 10.7554/eLife.28728 PMC5871355

[44] Stringer, C., Pachitariu, M., Steinmetz, N., Carandini, M. & Harris, K. D. High-dimensional geometry of population responses in visual cortex. *Nature* **571**, 361–365 (2019). 31243367 10.1038/s41586-019-1346-5 PMC6642054

