# Author Correction: Maturation and circuit integration of transplanted human cortical organoids

**DOI:** 10.1038/s41586-026-10943-4

**Published:** 2026-08-03

**Authors:** Omer Revah, Felicity Gore, Kevin W. Kelley, Jimena Andersen, Noriaki Sakai, Xiaoyu Chen, Min-Yin Li, Fikri Birey, Xiao Yang, Nay L. Saw, Samuel W. Baker, Neal D. Amin, Shravanti Kulkarni, Rachana Mudipalli, Bianxiao Cui, Seiji Nishino, Gerald A. Grant, Juliet K. Knowles, Mehrdad Shamloo, John R. Huguenard, Karl Deisseroth, Sergiu P. Paşca

**Affiliations:** 1 Department of Psychiatry and Behavioral Sciences, Stanford University, Stanford, CA USA; 2 Stanford Brain Organogenesis, Wu Tsai Neurosciences Institute and Bio-X, Stanford University, Stanford, CA USA; 3 Department of Bioengineering, Stanford University, Stanford, CA USA; 4 Department of Chemistry, Stanford University, Stanford, CA USA; 5 Stanford Behavioral and Functional Neuroscience Laboratory, Wu Tsai Neurosciences Institute, Stanford University, Stanford, CA USA; 6 Department of Comparative Medicine, Stanford University, Stanford, CA USA; 7 Department of Neurosurgery, Stanford University, Stanford, CA USA; 8 Department of Neurology and Neurological Sciences, Stanford, CA USA; 9 Howard Hughes Medical Institute, Stanford University, Stanford, CA USA

**Keywords:** Neuroscience, Developmental biology

Correction to: *Nature* 10.1038/s41586-022-05277-w Published online 12 October 2022

In the version of this article initially published, due to a figure preparation error, the mPFC panel in Fig. 5a was an inadvertent duplicate of the Fig. 5a A1 panel. The figure is now revised in the HTML and PDF versions of the article.

