# Supplementary material for: Maturation and circuit integration of transplanted human cortical organoids
Source: Nature. 2022 Oct 12;610(7931):319–26. doi: 10.1038/s41586-022-05277-w (PMC9556304; doi:10.1038/s41586-022-05277-w)
Supplement: Supplementary file 1 — Reporting Summary [file 41586_2022_5277_MOESM1_ESM.pdf]

nature portfolio

Corresponding author(s): Sergiu P. Pasca

Last updated by author(s): July 24, 2022

Reporting Summary

Nature Portfolio wishes to improve the reproducibility of the work that we publish. This form provides structure for consistency and transparency in reporting. For further information on Nature Portfolio policies, see our Editorial Policies and the Editorial Policy Checklist.

Statistics

For all statistical analyses, confirm that the following items are present in the figure legend, table legend, main text, or Methods section.

n/a	Confirmed	
☐	☒	The exact sample size (n) for each experimental group/condition, given as a discrete number and unit of measurement
☐	☒	A statement on whether measurements were taken from distinct samples or whether the same sample was measured repeatedly
☐	☒	The statistical test(s) used AND whether they are one- or two-sided<br>*Only common tests should be described solely by name; describe more complex techniques in the Methods section.*
☒	☐	A description of all covariates tested
☐	☒	A description of any assumptions or corrections, such as tests of normality and adjustment for multiple comparisons
☐	☒	A full description of the statistical parameters including central tendency (e.g. means) or other basic estimates (e.g. regression coefficient) AND variation (e.g. standard deviation) or associated estimates of uncertainty (e.g. confidence intervals)
☐	☒	For null hypothesis testing, the test statistic (e.g. F, t, r) with confidence intervals, effect sizes, degrees of freedom and P value noted<br>*Give P values as exact values whenever suitable.*
☒	☐	For Bayesian analysis, information on the choice of priors and Markov chain Monte Carlo settings
☒	☐	For hierarchical and complex designs, identification of the appropriate level for tests and full reporting of outcomes
☒	☐	Estimates of effect sizes (e.g. Cohen's d, Pearson's r), indicating how they were calculated

Our web collection on statistics for biologists contains articles on many of the points above.

Software and code

Policy information about availability of computer code

Data collection	Open Ephys (v0.4.4, in vivo extracellular electrophysiology recording acquisition, EEG acquisition), MATLAB R2019b (control of whisker deflection, data analysis, optogenetic behaviour), Prairie View (two-photon calcium imaging), Clampex (pClamp) 11.1 (Patch clamp recordings), FreezeFrame (fear conditioning), Paravision 6.0.1 (MR imaging); BZ-X analyzer (Keyence), Las-X (Leica) and Zen (Zeiss) software were used for acquiring immunoshistochemistry images.
Data analysis	MATLAB R2019b, ImageJ (image processing 1.53q and 2.0), GraphPad Prism 9.2.0, Kilosort2, Phy2, CNMF-E 1.1.2, Clampfit (pClamp) 10.6.2.2, Originlab 2021b SR2, Imaris 9.8.2 (MRI image processing and volume reconstruction), neuTube 1.0z, SimpleNeuriteTracer v4.0.12, CellRanger (v6.1.2, 10x Genomics), R (v4.1.2), Seurat (v4.1.1, R package), Libra (1.0.0, R package), ToppFun (toppgene.cchmc.org)<br>Code used to analyze snRNA-seq data is available for download from https://github.com/kkelley85/Transplant_organoid_snRNAseq.

For manuscripts utilizing custom algorithms or software that are central to the research but not yet described in published literature, software must be made available to editors and reviewers. We strongly encourage code deposition in a community repository (e.g. GitHub). See the Nature Portfolio guidelines for submitting code & software for further information.

Data

Policy information about availability of data

All manuscripts must include a data availability statement. This statement should provide the following information, where applicable:

- Accession codes, unique identifiers, or web links for publicly available datasets
- A description of any restrictions on data availability
- For clinical datasets or third party data, please ensure that the statement adheres to our policy

Data and custom code will be made available upon request.
Single nucleus RNA-seq data is available in GEO: accession number GSE190815.
The following public datasets were used for snRNA-seq analysis: human genome sequence information from Ensembl (http://ftp.ensembl.org/pub/release-98/fasta/homo_sapiens/dna/Homo_sapiens.GRCh38.dna.primary_assembly.fa.gz) and human gene annotation from GENCODE (http://ftp.ebi.ac.uk/pub/databases/gencode/Gencode_human/release_32/gencode.v32.primary_assembly.annotation.gtf.gz); rat genome sequence information from Ensembl (ftp://ftp.ensembl.org/pub/release-100/fasta/rattus_norvegicus/dna/Rattus_norvegicus.Rnor_6.0.dna.toplevel.fa.gz); Allen Brain Institute human adult snRNA-seq data from medial temporal gyrus and M1 cortex (https://portal.brain-map.org/atlases-and-data/rnaseq; accessed May 2022); human fetal cortical single-cell RNA-seq data (Polioudakis et al. obtained from http://solo.bmap.ucla.edu/shiny/webapp/ on April 2022; Trevino et al. was downloaded from GEO accession GSE162170); bulk RNA-seq data from developing human cortex generated by psychENCODE (http://development.psychencode.org/; accessed April 2022).

Human research participants

Policy information about studies involving human research participants and Sex and Gender in Research.

Reporting on sex and gender	Sample 1: Male (18 years old)<br>Sample 2: Female (3 years old)
Population characteristics	The two postnatal human tissue samples (age 3- and 18-year-old) were obtained from resection of frontal lobe cortex (middle frontal gyrus).
Recruitment	Samples were collected as part of surgeries for treating medically refractory epilepsy.
Ethics oversight	Human cerebral cortical tissue was obtained with informed consent under a protocol approved by the Stanford University Institutional Review Board.

Note that full information on the approval of the study protocol must also be provided in the manuscript.

Field-specific reporting

Please select the one below that is the best fit for your research. If you are not sure, read the appropriate sections before making your selection.

☒ Life sciences ☐ Behavioural & social sciences ☐ Ecological, evolutionary & environmental sciences

For a reference copy of the document with all sections, see nature.com/documents/nr-reporting-summary-flat.pdf

Life sciences study design

All studies must disclose on these points even when the disclosure is negative.

Sample size	Sample sizes were estimated empirically, based on previous studies (Birey et. al., Nature 2017; Marton et., Nature Neuroscience 2019; Pasca et al., Nature Medicine 2019, Khan et al., Nature Medicine 2020, Myura et al., Nature Biotechnology 2020).
Data exclusions	Animals with unintended experimental error were excluded based on poor hChR2 expression, fiber placement, or electrode positioning.<br>Rat nuclei, low quality nuclei, and putative doublets were removed from the analysis. Human nuclei were identified based on a conservative criteria of at least 95% of total mapped reads aligning to the human genome. Nuclei were considered low quality if they had low human genes per cell and high mitochondrial percentage. A detailed description of single nuclei exclusions and their rationale is shown in the Methods section. Neurons with poor, low quality recordings and/or morphological reconstructions were excluded. Otherwise, no data were excluded.
Replication	Data shown from representative experiments were repeated with similar results in at least 3 independent experiments, unless otherwise indicated by sample size. All attempts at replication were successful.
Randomization	The hiPSC lines used in each experiments are summarized in Supplementary Table 1.<br>Organoids were randomly selected for specific assays.<br>At least 2 hiPSC lines were used for experiments, except for the optotagging and 2P imaging experiments.
Blinding	Behaviour data was collected and analyzed by investigators blinded to the identity of the expressed gene (e.g. ChR2 or fluorescent protein).<br>The control and TS t-hCO comparisons were collected and analyzed independently by two experimentalists. Blinding was not relevant for other experiments.

Reporting for specific materials, systems and methods

We require information from authors about some types of materials, experimental systems and methods used in many studies. Here, indicate whether each material, system or method listed is relevant to your study. If you are not sure if a list item applies to your research, read the appropriate section before selecting a response.

Materials & experimental systems

n/a	Involved in the study
☐	☒ Antibodies
☐	☒ Eukaryotic cell lines
☒	☐ Palaeontology and archaeology
☐	☒ Animals and other organisms
☐	☒ Clinical data
☒	☐ Dual use research of concern

Methods

n/a	Involved in the study
☒	☐ ChIP-seq
☒	☐ Flow cytometry
☐	☒ MRI-based neuroimaging

Antibodies

Antibodies used

1) MC anti-c-Fos (rabbit, Abcam ab214672;[EPR20769]; 1:200)
2) PC anti-GFP (goat, Abcam ab6673 1:1000)
3) PC anti Netrin-G1a (Mouse, 1:100, R&D Systems, AF1166)
4) MC anti-CTIP2 (Rat, 1:300, Abcam, ab18465; [25B6])
5) PC anti-GFAP (Rabbit, 1:1,000, Dako, Z0334)
6) PC anti-GFP (Chicken, 1:1,000, GeneTex,GTX13970)
7) MC anti-human nuclear antigen-(HNA) (Mouse, 1:200, Abcam, ab191181; [235-1])
8) PC anti-NeuN (Rabbit, 1:500, Milipore, ABN78)
9) PC anti-PPP1R17 (Rabbit, 1:200, Atlas Antibodies, HPA047819)
10) MC anti-RECA-1 (Mouse, 1:50, Abcam, ab9774; [RECA-1])
11) PC anti-SCG2 (Rabbit, 1:100, Proteintech, 20357-1-AP)
12) PC anti-SOX9 (Goat, 1:500, R&D Systems, AF3075)
13) MC anti-STEM121 (Mouse, 1:200, Takara Bio, Y40410; [STEM121])
14) MC anti-CTIP2 (Rat, 1:300, Abcam, ab18465; [25B6])
15) MC anti-SATB2 (Mouse, 1:50, Abcam, ab51502; [SATBA4B10])
16) PC anti-GAD 65/67 (Rabbit, 1:400, Milipore, ABN904)
17) PC anti-PDGFRA (Rabbit,1:200, Santa Cruz, sc-338)
18) MC anti-NeuN (Mouse, 1:500, abcam ab104224 [1B7])
19) PC anti-iba1 (Goat, 1:100, abcam ab5076)

Validation

Validation and references on manufacturer's website.
For CTIP2, GFAP, GFP, PDGFRA, PPPlRl 7 and SOX9 see also Andersen et al., Cell 2020, Trevino et al Science., 2020 and Trevino et al., Cell 2021.

Other antibodies have been used and validated in other studies under manufacturer website as follows:

Anti-GFP antibody (goat, Abcam ab6673 1:1000) - 391 studies
Anti-c-Fos antibody (rabbit, Abcam ab214672;[EPR20769]; 1:200) - 4 studies
Anti-human nuclear antigen-(HNA) (Mouse, 1:200, Abcam, ab191181; [235-1])- 33 studies
anti-NeuN (Rabbit, 1:500, Milipore, ABN78) - 393 studies
anti-RECA-1 (Mouse, 1:50, Abcam, ab9774; [RECA-1])- 89 studies
anti Netring1a (Mouse, 1:100, R&D Systems, AF1166) - 3studies
anti-SCG2 (Rabbit, 1:100, Proteintech, 20357-1-AP) - 3 studies
anti-STEM121 (Mouse, 1:200, Takara Bio, Y40410; [STEM121]) - 5 studies
anti-SATB2 (Mouse, 1:50, Abcam, ab51502; [SATBA4B10]) - 206 studies
anti-GAD 65/67 (Rabbit, 1:400, Milipore, ABN904) - 20 studies
anti-NeuN (Mouse, 1:500, abcam ab104224 [1B7]) - 331 studies
anti-iba1 (Goat, 1:100, abcam ab5076) - 844 studies

Eukaryotic cell lines

Policy information about cell lines and Sex and Gender in Research

Cell line source(s)

2242-1 Stanford IRB; 8119-1 UCLA IRB;1208-2 UCLA IRB; 1205-4 NIH IRB; CW10177 UCSF IRB;CW30270 Stanford IRB; 0524-1 9862-2 Stanford IRB; 7643-6 Stanford IRB; 8303-4 Stanford IRB

Authentication

hiPSC lines were assessed for genomic integrity by SNP microarray "GSAMD-24v2--0"

Mycoplasma contamination

Cell lines were tested for Mycoplasma contamination and tested negative

Commonly misidentified lines (See ICLAC register)	No commonly misidentified lines were used

Animals and other research organisms

Policy information about studies involving animals; ARRIVE guidelines recommended for reporting animal research, and Sex and Gender in Research

Laboratory animals	FOXN1-/- male and female rats between P3 and 9 months of age were used in this study
Wild animals	No wild animals were used in this study
Reporting on sex	Both male and female rats were used for experiments. Male and female animals were randomly assigned to experiments.
Field-collected samples	No field samples were collected in this study
Ethics oversight	All experiments involving human cells complied with all relevant guidelines and regulations. Human donors in this study consented to the use of their cells to generate hiPSC and derived cells. The source of the cells and their institutional approvals are listed in Supplementary Table 1. This study also benefited from a consultation with the Stanford Center for Law and the Biosciences on the ethical aspects of the work as part of the Stanford Big Idea Project on Brain Organogenesis (Stanford Wu Tsai Neurosciences Institute).<br>Approval for transplantation of hCO into rats was obtained from the Stanford Laboratory Animal Care (APLAC) Research Compliance Office. No discernible locomotor or memory deficits were detected in transplanted animals and their well-being was monitored throughout.<br>Surgical neural tissue samples were obtained with approval from the Stanford University Institutional Review Board (IRB).

Note that full information on the approval of the study protocol must also be provided in the manuscript.

Clinical data

Policy information about clinical studies

All manuscripts should comply with the ICMJE guidelines for publication of clinical research and a completed CONSORT checklist must be included with all submissions.

Clinical trial registration	*Provide the trial registration number from ClinicalTrials.gov or an equivalent agency.*
Study protocol	*Note where the full trial protocol can be accessed OR if not available, explain why.*
Data collection	*Describe the settings and locales of data collection, noting the time periods of recruitment and data collection.*
Outcomes	*Describe how you pre-defined primary and secondary outcome measures and how you assessed these measures.*

Magnetic resonance imaging

Experimental design

Design type	Anatomical brain structures in anesthetized rats
Design specifications	not applicable
Behavioral performance measures	not applicable

Acquisition

Imaging type(s)	Structural
Field strength	7T
Sequence & imaging parameters	Axial 2D Turbo-RARE (TR=2500 ms, TE=33 ms, 2 averages) 16 slice acquisitions were performed with 0.6-0.8 mm slice thickness, with 256x256 samples. Signal was received with a 2 cm inner-diameter quadrature transmit-receive volume radio frequency coil (Rapid MR international LLC)
Area of acquisition	Whole rat brain

Diffusion MRI ☐ Used ☒ Not used

Preprocessing

Preprocessing software	not applicable

Normalization	not applicable
Normalization template	not applicable
Noise and artifact removal	not applicable
Volume censoring	not applicable

Statistical modeling & inference

Model type and settings	not applicable
Effect(s) tested	not applicable

Specify type of analysis: ☒ Whole brain ☐ ROI-based ☐ Both

Statistic type for inference (See Eklund et al. 2016)	not applicable
Correction	not applicable

Models & analysis

n/a	Involved in the study
☒	☐ Functional and/or effective connectivity
☒	☐ Graph analysis
☒	☐ Multivariate modeling or predictive analysis
